# The heterotrimeric G protein β subunit *RGB1* is required for seedling formation in rice

**DOI:** 10.1186/s12284-019-0313-y

**Published:** 2019-07-18

**Authors:** Yun Gao, Houwen Gu, Mamotshewa Leburu, Xuhui Li, Yan Wang, Jiayan Sheng, Huimin Fang, Minghong Gu, Guohua Liang

**Affiliations:** Jiangsu Key Laboratory of Crop Genetics and Physiology/Key Laboratory of Plant Functional Genomics of the Ministry of Education/Jiangsu Key Laboratory of Crop Genomics and Molecular Breeding, Jiangsu Co-Innovation Center for Modern Production Technology of Grain Crops, Agricultural College, Yangzhou University, Yangzhou, 225009 China

**Keywords:** Heterotrimeric G protein, RGB1, Early postgermination development, Cell death, Rice

## Abstract

**Background:**

The heterotrimeric G protein β subunit RGB1 plays an important role in plant growth and development. However, the molecular mechanisms underlying the regulation of rice growth by RGB1 remain elusive.

**Results:**

Here, the *rgb1* mutants *rgb1–1* (+ 1 bp), *rgb1–2* (− 1 bp), and *rgb1–3* (− 11 bp) were isolated using the CRISPR/Cas9 system, and they were arrested at 1 day after germination and ultimately exhibited seedling lethality. The dynamic anatomical characteristics of the embryos of the *rgb1* seedlings and WT during early postgermination and according to TUNEL assays showed that the suppressed growth of the *rgb1* mutants was caused by cell death. In addition to the limited shoot and root development, the development of the embryo shoot-root axis was suppressed in the *rgb1* mutants. *RGB1* was expressed mainly in the root epidermal and vascular tissues of the embryo. Moreover, transcript profiling analysis revealed that the expression of a large number of auxin-, cytokinin-, and brassinosteroid-inducible genes was upregulated or downregulated in the *rgb1* mutant compared to the wild type during seedling development.

**Conclusions:**

Overall, the *rgb1* mutants provide an ideal material for exploring the molecular mechanism underlying rice seedling formation during early postgermination development by G proteins.

**Significance statement:**

The heterotrimeric G protein β subunit RGB1 acts as a crucial factor in promoting early postgermination seedling development in rice.

**Electronic supplementary material:**

The online version of this article (10.1186/s12284-019-0313-y) contains supplementary material, which is available to authorized users.

## Background

G proteins play crucial roles as signal transducers in the regulation of a variety of growth and development processes in higher eukaryotes. G proteins can be classified into heterotrimeric GTP-binding proteins, small GTPases, and other unconventional Gα proteins, such as XLGs (Ding et al. 2008; Wu et al. 2018). Heterotrimeric G proteins, which are composed of α, β, and γ subunits, generally refer to the G proteins. In contrast to those in humans, G protein subunits in plants are simple and are usually encoded by single gene or a few genes. The GPA1 and AGB1 subunits are encoded by a single gene each, while Gγ1, Gγ2, and Gγ3 are encoded by three genes (*AGG1*, *AGG2*, and *AGG3*) in *Arabidopsis* (Temple and Jones 2007). The fully sequenced rice genome contains only one conventional Gα (*RGA1*), one Gβ (*RGB1*), and five Gγ homologs, which include one type-A Gγ1 (*RGG1*), one type-B Gγ2 (*RGG2*), and three type-C Gγ3 genes (homologous to *AGG3*): *Panicle Erect9–1*/*DENSE PANICLE 1/DENSE AND ERECT PANICLE1* (*qPE9–1/DN1/DEP1*), *GRAIN SIZE 3* (*GS3*), and a Gγ type-C2 gene (*OsGGC2*) (Huang et al. 2009; José Ramón 2012; Taguchi-Shiobara et al. 2011; Zhou et al. 2009). In plants, Gα spontaneously releases GDP and forms a stable GTP-bound state, after which Gβγ dissociates from the Gα protein (Jones et al. 2011; Urano et al. 2012). Gα·GTP and the free Gβγ complex interact separately with a variety of downstream effectors to initiate signals for many cellular processes (Mccudden et al. 2005). Studies of G proteins in *Arabidopsis thaliana* and in rice by mutations and in transgenic plants indicate that G proteins mediate multiple developmental processes (Urano et al. 2013). However, the exact function of RGB1 remains elusive in rice.

In *Arabidopsis*, hypocotyls of the *gpa1* mutant seedlings are short, and the typical hooks are open when grown in the dark (Ullah et al. 2001). Compared with *gpa1* mutants, *agb1* mutants exhibit shorter hypocotyls and reduced epidermal cell numbers (Ullah et al. 2003). The G protein γ subunits AGG1, AGG2, and AGG3 provide functional selectivity in Gβγ dimer signaling, and the triple gamma mutant (*agg1agg2agg3*) exhibits the phenotype of the *agb1* mutant (Thung et al. 2012; Trusov et al. 2007). In rice, *RGA1* is involved in gibberellin signal transduction, and the loss-of-function allele was isolated from a severe dwarf-type mutant (Ashikari et al. 1999). *GS3* mainly regulates grain size, and *qPE9–1/DEP1* mainly regulates panicle length (Huang et al. 2009; Mao et al. 2010; Zhou et al. 2009). *RGB1* knock-down lines in both WT and *d1* (*rga1*) backgrounds were dwarf, and subsequent lines were shorter, which suggests that RGB1 is a positive regulator of cell proliferation (Utsunomiya et al. 2011). Transgenic plants with greatly decreased expression of *rgb1* were not obtained, which hints at the lethal impact of complete *rgb1* mRNAi suppression (Utsunomiya et al. 2012).

Plant survival begins with seed germination and the formation of seedlings under suitable environmental conditions. The mature seed consists of the embryo, endosperm, and seed coat, which are formed during embryogenesis in higher plants. In *A. thaliana*, the inner seed space is mostly occupied by the embryo and nutrients that are mainly stored in the cotyledons. The structure of a mature rice seed is very different from that of a mature *Arabidopsis* seed, and the nutrients in the grains accumulate largely in the endosperm (Keith et al. 1994; Olsen 2004). During rice seed germination, active gibberellin (GA) biosynthesis first commences in the embryo, and GAs are then transported from the embryo to the aleurone layer through the scutellum to trigger the expression of α-amylase gene transcription (Fincher 1989; Gubler et al. 1995). Hydrolases are secreted from the aleurone layer into the endosperm and cause the degradation of the stored starch into soluble sugars, which are then transported to the scutellum (Akazawa and Hara-Mishimura 1985; Beck and Ziegler 1989). The nutrients in the scutellum flow into the vascular bundles, which connect the roots and shoots and serve as the main channels for the upward and downward transport of water and nutrients in rice.

Programmed cell death (PCD) is an orderly process that occurs throughout development in animals and plants. For plants to survive and develop properly, PCD is an important response strategy for the adaptation to various internal and external cues. Both germination and seedling formation rely on a continuous remobilization of nutrients, which is supported by cell degeneration. In maize, similar to CT2, ZmXLG1, ZmXLG3a, and ZmXLG3b are able to interact with Gβγ. All *Zmxlg* triple mutant plants show a striking developmental arrest, and they are lethal at the seedling stage because of cell death (Wu et al. 2018). In rice, cell death is induced by ethylene and mediated by hydrogen peroxide (H_2_O_2_), which indicates that rice Gα is essential in epidermal cell death signaling and acts downstream of ethylene and H_2_O_2_ (Sauter 2009).

Various signals such as phytohormones, light, oxygen, sucrose, and developmental factors modulate seedling formation in plants. G proteins have been shown to interact with the BR receptors BRI1 and BAK1 to regulate sugar-responsive plant development in *Arabidopsis* (Peng et al. 2018). In rice, a member of the heterotrimeric G protein βγ dimer, DEP1/qPE9–1, interacts with MAD proteins to regulate the auxin efflux carrier *OsPIN1a* and the response factor genes *OsARF9* and *OsARF14* in developing panicles and spikelet hulls (Liu et al. 2018). Crosstalk between G proteins and several hormone signaling pathways has also been reported to be involved in plant growth (Chakravorty and Botella 2007; Tsugama et al. 2013; Ullah et al. 2001).

The loss-of-function G protein mutants *rga1*, *gs3*, and *qpe9–1*/dep1 were found to be natural rice variants, while those of the mutants *rgb1*, *rgg1* and *rgg*2 have not been obtained. After several years of effort, loss-of-function *rgb1* mutants were isolated and have been used to explore the exact function of RGB1 during early postgermination development in rice. Here, we describe the dynamic morphological and anatomical characteristics of the embryos during the postgermination development of *rgb1* mutants. The results suggest that RGB1 is an important factor in promoting seedling growth and development during early postgermination in rice.

## Results

### Identification and characterization of *rgb1* mutants

**Fig. 1:**
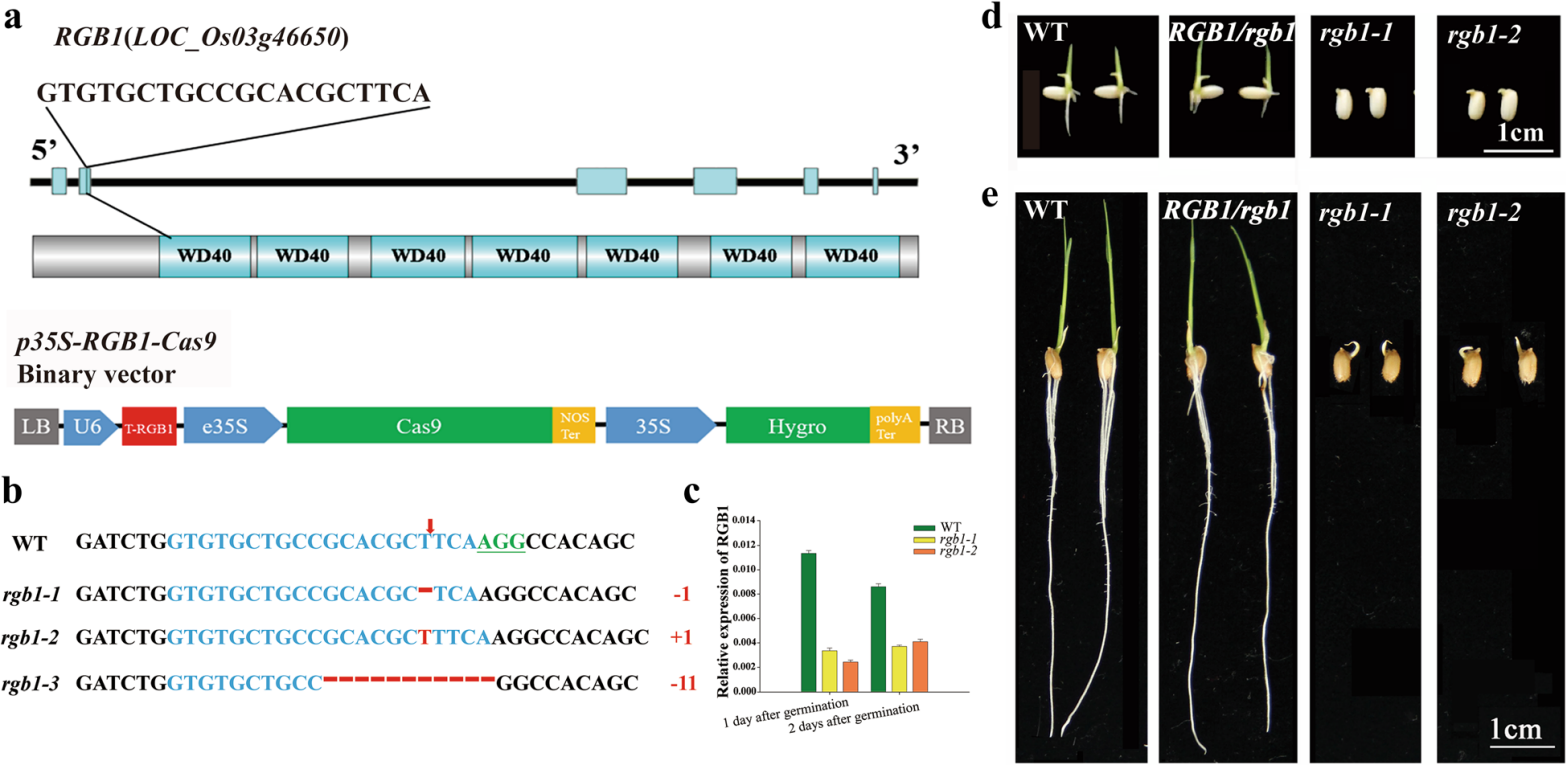
Characterization of the *rgb1* mutants generated using CRISPR/Cas9 in rice. **a** Schematic diagram of the genomic region and the predicted protein structure of RGB1 and the target guide sequences are labeled in black uppercase letters. The structure of the *p35S-RGB1_Cas9* vector is shown at the bottom of the panel. **b** Sequence alignment of the *rgb1* mutants showing altered bases compared to the reference genomic *RGB1* sequence. The red arrow indicates the cleavage sites. The targeted sequence is highlighted in blue, and the protospacer adjacent motif (PAM) sequences are marked in green and underlined. Indels are indicated by red letters (insertions) or dashes (deleted nucleotides). **c** Real-time RT-PCR analysis of *RGB1* transcripts in the embryos of the WT and *rgb1* mutant lines during the early stages of postgermination growth. The PCR signals were normalized to those of actin transcripts. The data are presented as the means±SDs. (*n* = 3) (**d-e**) Comparison of seedlings of the *rgb1* mutant, *RGB1/rgb1* mutant and wild type. **d** Germinated dehulled seeds growing on filter paper atop wet sand for 5 days after germination. **e** Germinated seeds growing on plates containing distilled water for 5 days after germination. The white scale bars represent 1 cm

To identify the lethal stage of the *rgb1* mutants and explore the exact function of RGB1, the CRISPR/Cas9 genome editing system was used to knock out *RGB1* in the ‘Zhonghua11’ background (Fig. 1a). *RGB1* loss-of-function mutants were not obtained in the T0 and T1 generations in the experimental fields after we tested the target DNA sequences in all the surviving seedlings at the four-leaf stage (Additional file 7: Table S2). However, heterozygous mutant plants could be identified by either conventional sowing or direct hill seeding. The loss of function of RGB1 was expected to be lethal in ‘Zhonghua 11’ before the four-leaf stage. To verify the stage in which the seedlings die, the viability of pollen from the different *RGB1* heterozygous mutant lines (SG948–6, SG948–8 and SG948–13) was first examined; the pollen was found to be viable in these lines (Additional file 1: Figure S1a-d). More than 90% of the seeds showed good grain plumpness, and normal shoot and root structures had developed from the embryos on day 18 after flowering (Additional file 1: Figure S1e-h). To identify the genotype, seeds and grains (dehulled) from the SG948–6 and SG948–8 lines in the T2 generation were germinated and grown for 1 week. The germination efficiency of the dehulled grains and seeds ranged from 58.33% to 65.0% and from 72.57% to 75.69%, respectively (Table 1). The segregation ratios of the four heterozygous lines for the germinated and nongerminated seeds fit a 3:1 ratio well (χ^2^ = 0.16, 0.29, 0.03, 0.29 < χ^2^_0.05,1_ = 3.84). These results suggest that the germination differences among the RGB1 heterozygous mutants are controlled by a single gene.

**Table 1:** Germination efficiency in T2-generation *p35S-RGB1-Cas9* (Aa) and WT (Aa, RGB1 heterozygous mutants). (NG: Number of seeds, NGS: Number of germinated seeds, NNGS: Number of nongerminated seeds, GE: Germination efficiency)

	WT		SG948–6 (Aa)		SG948–8 (Aa)	
(a)	SG948–8-5	SG948–6-9	SG948–6-1	SG948–6-11	SG948–8-1	SG948–8-8
NS	40	40	40	40	40	40
NGS	38.3 ± 0.6	38.7 ± 1.5	26.0 ± 2.6	23.7 ± 1.5	24.7 ± 3.5	23.3 ± 2.1
NNGS	1.7 ± 0.6	1.3 ± 1.5	14 ± 2.6	16.3 ± 1.5	15.3 ± 3.5	16.7 ± 2.1
GE (%)	95.83 ± 1.44	96.67 ± 3.82	65.0 ± 6.61**	59.17 ± 3.82**	61.67 ± 8.78**	58.33 ± 5.20**
Dehulled seeds were used. ** indicates a significant difference compared with the wild type at *p* < 0.01						
	WT		SG948–6 (Aa)		SG948–8 (Aa)	
(b)	SG948–8-5	SG948–6-9	SG948–6-1	SG948–6-11	SG948–8-1	SG948–8-8
NS	96	96	96	96	96	96
NGS	96.0 ± 0.0	96.0 ± 0.0	72.7 ± 2.5	69.7 ± 2.1	70.3 ± 3.1	69.7 ± 2.5
NNGS	0.0 ± 0.0	0.0 ± 0.0	23.3 ± 2.5	26.3 ± 2.1	25.7 ± 3.1	26.3 ± 2.5
GE (%)	100.0 ± 0	100.0 ± 0	75.69 ± 2.62**	72.57 ± 2.17**	73.26 ± 3.18**	72.57 ± 2.62**
Rice seeds were used. ** indicates a significant difference compared with the wild type at *p* < 0.01						

Genomic DNA was extracted from individual embryos of nongerminated seeds (those seeds that had germinated but lacked roots and shoots), and the DNA fragment that included the target sequence was amplified and sequenced. There was a single mutation in the homozygous mutants of lines SG948–6 (+ 1 bp) and SG948–8 (− 1 bp), but there were two mutation sites in line SG948–13 (+ 1 bp, − 11 bp) (Additional file 7: Table S2). Three types of *RGB1* loss-of-function mutant lines, named *rgb1–1* (+ 1 bp), *rgb1–2* (− 1 bp) and *rgb1–3* (− 11 bp), were identified (Fig. 1b, Additional file 2: Figure S2a-d). The SG948–6 and SG948–8 lines were used to perform real-time PCR assays to precisely name the *rgb1* mutants. The *RGB1*-Cas9 target sequence was used for the forward primer for real-time PCR, and the expression of *RGB1* could not be detected in the *rgb1–1* and *rgb1–2* mutants but was detected in the WT (Additional file 2: Figure S2e). Expression of *RGB1* in the embryos, including the *RGB1*-Cas9 target sequence, was significantly downregulated in the *rgb1* mutants compared with the WT (Fig. 1c). The relative expression of six other G protein family member subunit genes is shown in Additional file 2: Figure S2f-h. The expression levels of *RGG2* during the early postgermination stage were significantly higher than those of the other G protein family genes, and *RGA1* and *RGG2* were significantly upregulated in the *rgb1* mutants. *RGG1*, *qPE9–1*, *GS3* and *OsGGC2* showed low expression in both the WT and *rgb1* mutants. All three mutants were able to germinate but did not produce roots or shoots (Fig. 1d, e), even in seedlings at 5 days after germination, suggesting that the *RGB1* loss-of-function mutants are early postgermination lethal.

### Expression patterns of *RGB1* in germinated seeds and young seedlings

**Fig. 2:**
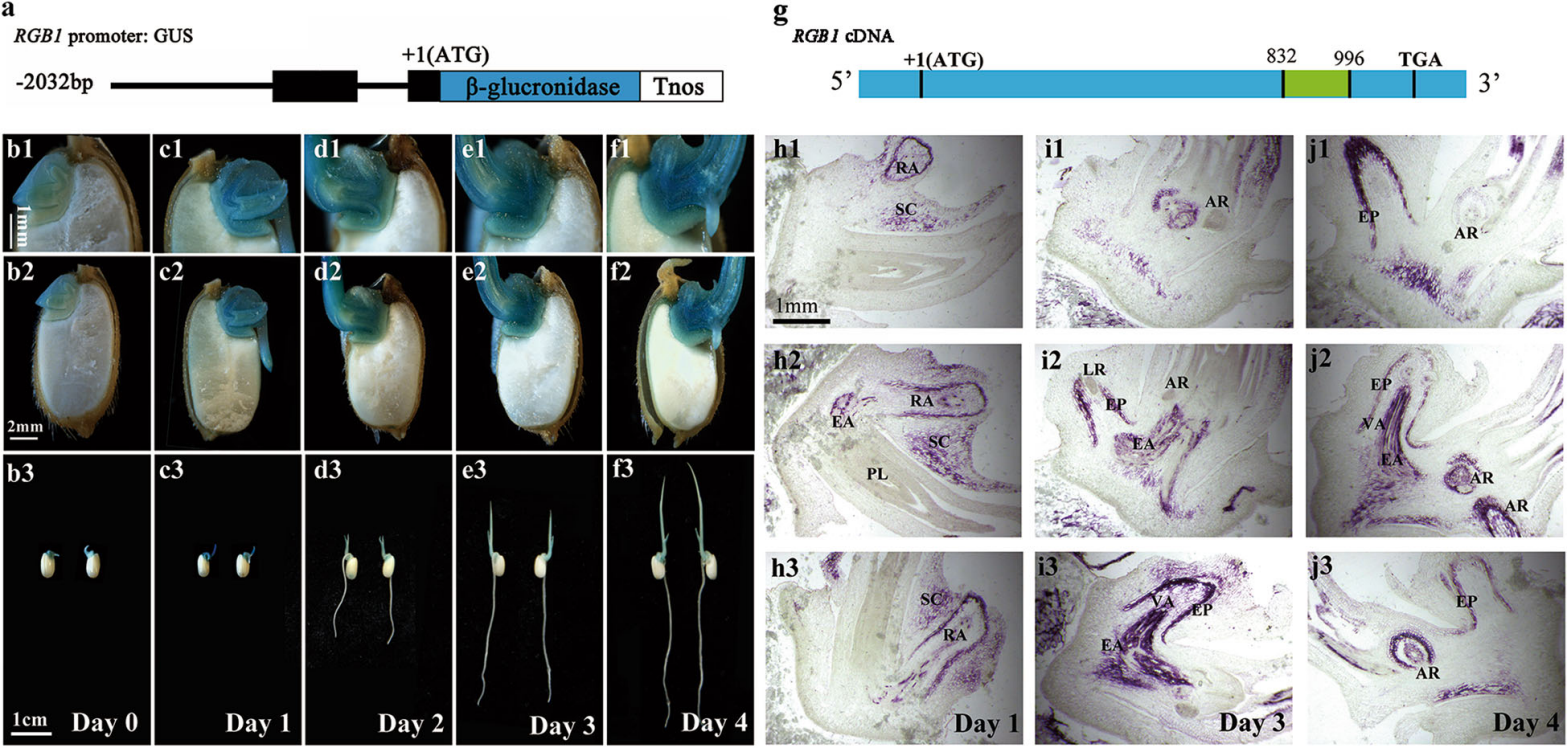
Expression pattern analysis of *RGB1* in the embryo during early postgermination development in rice. **(a)** RGB1 promoter::GUS fusion gene. The 2.032-kb promoter region and the 105-bp fragment after ATG of RGB1 were used to drive the expression of the GUS gene. **(b-f)** Expression of the RGB1:GUS construct in median longitudinal sections of embryos. RGB1 was expressed in geminated seeds and young seedlings, strongly in the shoot-root axis. **(b1-b3)** Germinated seeds, **(c1-c3)** 1-day-old seedlings, **(d1-d3)** 2-day-old seedlings, **(e1-e3)** 3-day-old seedlings, **(f1-f3)** 4-day-old seedlings. **(g)** Location of the probe used for in situ hybridization of the *RGB1* cDNA sequence (the fragment is shown in green). **(h-j)** In situ hybridization detection of *RGB1* transcripts in rice embryos after germination. **(h1-h3)** Median longitudinal sections of 1-day-old wild-type seedlings. RA, radicle. SC, scutellum. EA, embryonic axis. PL, plumule. **(i1-i3)** Median longitudinal sections of 3-day-old wild-type seedlings. AR, adventitious root. EP, epidermis. EA, embryonic axis. VA, vascular tissue. LR, lateral root. **(j1-j3)** Median longitudinal sections of 4-day-old wild-type seedlings. AR, adventitious root. EP, epidermis. EA, embryonic axis. VA, vascular tissue. LR, lateral root

To examine the expression patterns of *RGB1*, promoter-reporter gene fusion studies were performed during seed germination and early postgermination (Fig. 2a). GUS staining patterns at germination were analyzed after the seeds were soaked in distilled water for 3 days and the sheaths had developed. GUS reporter gene activity was detected in the scutellum, germ, and radicle and was slightly stronger in the vascular tissues (Fig. 2b1, b2, b3). For the 24-h postgermination seedlings, the root length ranged from 2 mm to 7 mm, and the sheath had turned green in color. The expression of *RGB1* became stronger in the embryo and primary root compared with the other tissues and was the strongest in vascular tissue (Fig. 2c1, c2, c3). The roots grew rapidly, and the first leaf developed in the 48 h postgermination seedlings. The expression of *RGB1* in the roots was not detected but was detected in the sheath and embryo and especially in the vascular tissues (Fig. 2d1, d2, and d3). The growth of the shoot-borne roots and lateral roots of seedlings initiated between 72 and 96 h post germination, and the expression of *RGB1* was most intense in the vascular tissues of the embryos (Fig. 2e1-e3, f1-f3).

In situ hybridization was also used to detect the expression of *RGB1* mRNA in the WT seedlings (Fig. 2g). On the first day after germination, *RGB1*-specific mRNA was mainly detected in the primary root, the scutellum around it and the embryonic axis in the embryo (Fig. 2 h1, h2, h3). The *RGB1* expression became strong in the roots and embryonic axis in 3-day-old seedlings. However, weak expression was detected in the seedling shoots, but intense expression was detected in the vascular tissues and root epidermis (Fig. 2i1, i2, i3). Moreover, *RGB1* expression was detected in the adventitious roots of 4-day-old seedlings and showed a similar expression pattern in primary roots (Fig. 4j1, j2, j3).

### *RGB1* regulates callus growth in culture, and supplementation of media with different hormones cannot rescue the abnormal seedling phenotype

**Fig. 3:**
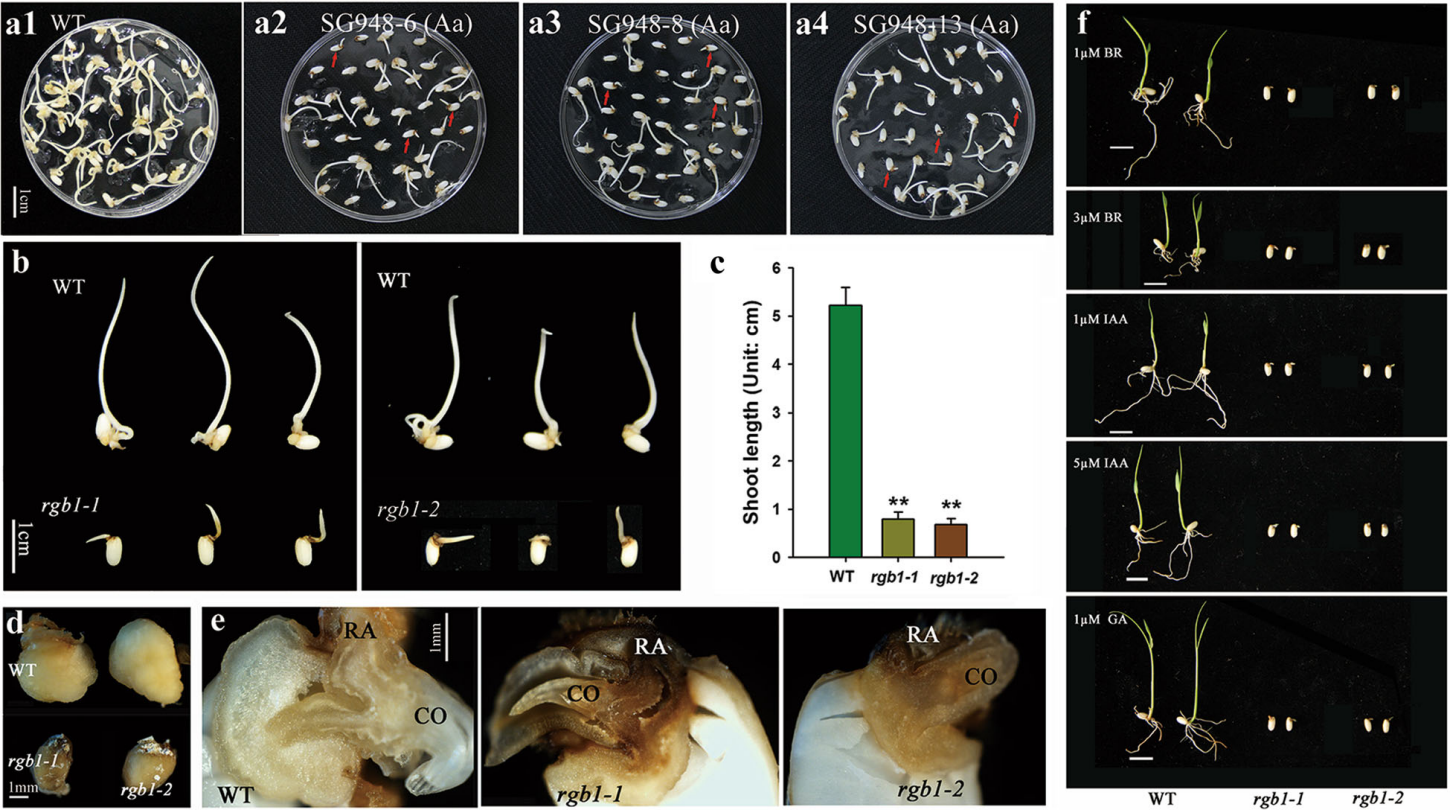
Callus induction from the mature seeds after 10 days and the different hormone treatments of *rgb1* mutants, RGB1 heterozygous mutants, and WT. **(a)** Mature seeds of the RGB1 heterozygous mutant lines and WT cultured on callus preinduction media (N6D2). **(a1)** WT control, **(a2)** SG948–6 (Aa), **(a3)** SG948–8 (Aa), **(a4)** SG948–13 (Aa) (Aa indicates heterozygous *RGB1/rgb1* mutants). **(b)** Calli induced from the *rgb1–1* and *rgb1–2* mutants compared with WT. **(c)** Statistical analysis of shoot lengths of the WT and the *rgb1* mutants shown in **(b)**. The data are the means ± SDs (*n* = 10; **p < 0.01). **(d)** Comparison of the morphology of calli from the WT and the *rgb1* mutants. **(e)** Median longitudinally dissected embryos of WT and *rgb1* mutants (CO, coleoptile; RA, radicle). **(f)** Phenotypic observations of 1-week-old seedlings of the *rgb1* mutants and WT under 1 μM BRs, 3 μM BRs, 1 μM IAA, 5 μM IAA, and 1 μM GA. Scale bar, 1 cm

A method in which the embryos from mature seeds are used to induce callus to generate transgenic plants by Agrobacterium-mediated transformation was used to complement the *rgb1* mutants (Hiei et al. 2008). Fresh, healthy calli were produced from WT seeds, while brown embryos and short shoots were observed from heterozygous *RGB1* mutant seeds except for normal calli (Fig. 3a1, a2, a3, a4). The abnormal seeds with brown embryos and short shoots among the heterozygous *RGB1* mutant lines were verified as homozygous *rgb1* mutants. The wild type formed calli and elongating shoots after the mature seeds had been in culture for 10 days. However, the development of calli induced from the *rgb1* mutant embryos was suppressed, and the shoot elongation was significantly inhibited compared to that of the WT (Fig. 3b, c, d). The radicles of the *rgb1* mutants were brown, while the WT radicles were much lighter in color (Fig. 3e). These results suggest that *RGB1* is a key regulator of callus formation in rice.

The addition of exogenous auxin was able to partially rescue the overall height and abnormal development of the primary, lateral, and adventitious roots of the transgenic plants. As shown in Additional file 3: Fig. S3A, the expression of RGB1 is induced by NAA in both the shoots and roots. Exogenous IAA at different concentrations was used to treat the rgb1 mutants after germination, but this treatment did not affect the abnormal shoots and roots (Fig. 3f). The content of IAA in the embryos of the rgb1 mutants was significantly lower than that in the 2-day-old WT seedlings but was significantly higher than that in the embryos of the 3-day-old WT seedlings (Additional file 3: Fig. S3b). In addition to IAA, the contents of gibberellin, abscisic acid, cytokinin, and BRs were also measured in the embryos of the rgb1 mutants and in the in 3-day-old control seedlings (Additional file 3: Fig. S3c-g). IPA was not detected in the rgb1 mutants but was detected at low levels (~0.05 ng/g) in the WT, and the content of BL in the rgb1 mutant was >10-fold that of the control embryos of the 3-day-old seedlings. The shoots and roots also did not grow in the rgb1 mutants when treated with BRs or GA3 (Fig. 3f). These results indicate that rgb1 is related to endogenous hormone disorders in the embryos, which represses rice seedling growth, and that supplementation of media with different hormones cannot rescue the abnormal seedling phenotype.

### Embryo cell death of the *rgb1* mutant is impaired

**Fig. 4:**
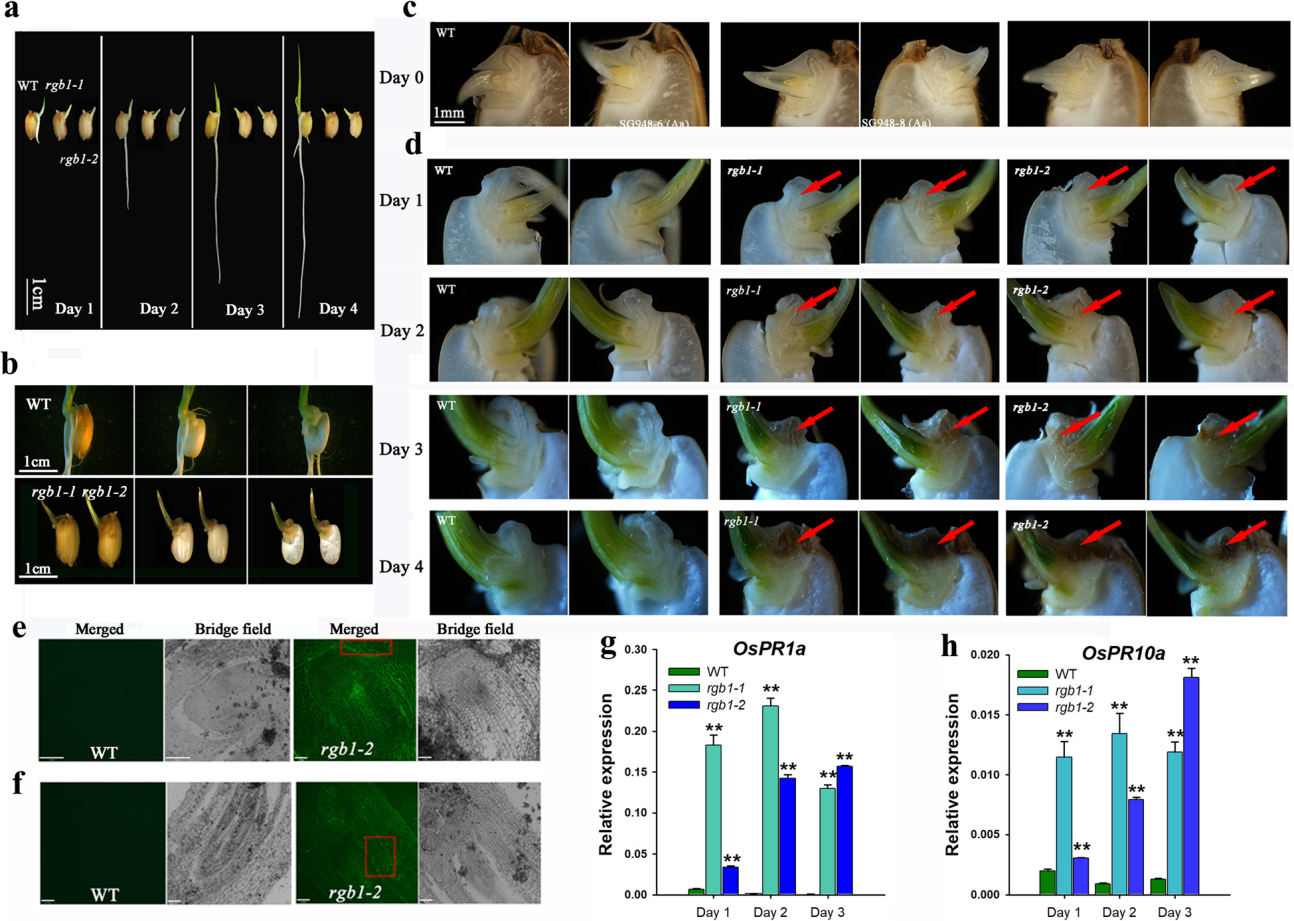
Embryo cell death of the *rgb1* mutant is impaired. **a** Phenotypes of WT and *rgb1* mutant seedlings at one to 4 days after germination. **b** Median longitudinal sections of 7-day-old *rgb1* mutant and wild-type seedlings. **c** Median longitudinal sections of WT and *RGB1/rgb1* heterozygous seeds at 0 days after germination. The embryo radicle color was the same as of the WT at 0 days after germination. **d** Median longitudinal sections of WT and *rgb1* mutant embryos in 1- to 4-day-old seedlings. From day 1 to day 4, the color of the *rgb1* mutant radicles changed from white to yellowish to brown and then to dark brown, while the WT radicles were always white. (The red arrow indicates the location of the radicle in the embryo.) **e, f** TUNEL assay of the 4-day-old embryos of the *rgb1–2* mutant and 1-day-old WT embryos. TUNEL-positive cells showed bright green signal spots in the boxed areas. Scale bar, 70 μm. **g, h** Transcript levels of two immune marker genes, *OsPR1a* and *OsPR10a*, in the embryos of 1-, 2- and 3-day-old seedings of the WT and the *rgb1* mutants. **h** *OsPR1a*, **i** *OsPR10a*. The data are means ± SDs (n = 3; **p < 0.01)

Seedlings of the two kinds of *rgb1* mutant lines stopped growing on the first day after germination (Fig. 4a). To further analyze the phenotype of the *rgb1* mutants, we dissected embryos of one-week-old mutants longitudinally and found that the growth of the lateral organs that were differentiated from the apical meristem was inhibited, in contrast to that of the WT. The shoots and roots of the *rgb1* mutants also stopped growing on the first day after germination, and the radicle turned brown (Fig. 4b). The germinated seeds were defined as 0-day-old seedlings, and 1- to 5-day-old seedlings were those at one to 5 days after germination (Additional file 4: Figure S4). The seeds of the *rgb1* mutants were dissected longitudinally at 0, 1, 2, 3 and 4 days after the seeds of the *RGB1* heterozygous mutant lines had germinated, and the embryos were observed and imaged with a macroscopic zoom microscope (Olympus). The embryo phenotype could not be clearly distinguished from that of the RGB1 heterozygous mutants at 0 h after germination when 20 seeds were longitudinally dissected and examined. The embryo radicle color was the same as that of the WT at 0 days after germination (Fig. 4c). However, from day 1 to day 4, the color of the *rgb1* mutant radicles changed from white to yellowish to brown and then to dark brown, while the WT radicles were always white (Fig. 4d). The fact that the shoots and roots of the *rgb1* mutant stopped growing on day 1 after germination indicated that *RGB1* is indispensable during early postgermination development in rice.

The gradual changes in color of the radicle of the *rgb1* mutants suggested that the lethality might be caused by PCD. To test our hypothesis, a TdT-mediated d-UTP nick-end labeling (TUNEL) assay was used to determine whether cell death was involved in the *rgb1* mutants. As shown in Fig. 4e and f, the *rgb1* mutant had bright green spot signals in the boxed areas of the embryo compared to the control, suggesting that the mutant was undergoing cell death. We measured the expression of two immunity marker genes, *OsPR1a* and *OsPR10a*, and found that both were significantly upregulated in the *rgb1* mutants from 1 to 3 days after germination (Fig. 4g, h), indicating that the lethality may be due to the overactivation of the plant immune system.

### *RGB1* regulates the development of the shoot-root axis during the early postembryonic stage

**Fig. 5:**
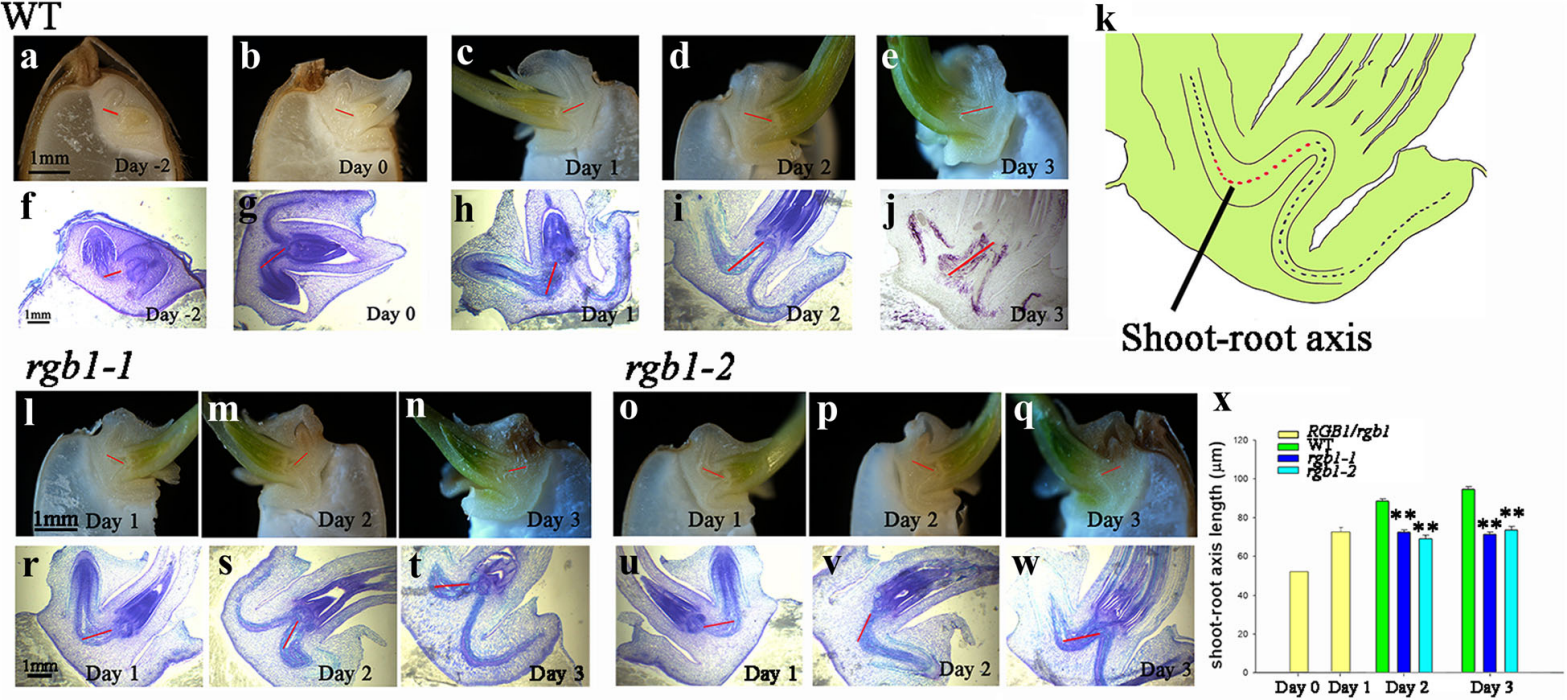
*RGB1* positively regulates shoot-root axis development in embryos during the early postgermination stage. (The red line indicates the location of the shoot-root axis.) **a-e** Median longitudinal sections and sections of WT embryos (**a**) soaked in distilled water for 1 day (day − 2), **b** soaked in distilled water for 3 days (day 0), **c** at 1 day after germination, **d** at 2 days after germination, and (**e**) at 3 days after germination. **f-j** Median longitudinal sections and sections of WT embryos (**f**) soaked in distilled water for 1 day (day − 2), **g** soaked in distilled water for 3 days (day 0), **h** at 1 day after germination, **i** at 2 days after germination, and (**j**) at 3 days after germination. **k** Diagram showing the location of shoot-root axis length (red dotted line). **l-q** Median longitudinal sections of *rgb1* embryos at 1, 2, and 3 days after germination. **r-w** Median longitudinal sections of *rgb1* embryos at 1, 2, and 3 days after germination. **x** Shoot-root axis lengths of the *rgb1* mutant, *RGB1* heterozygote, and WT at 0, 1, 2, and 3 days after germination. The data are means ± SDs (n = 10; **p < 0.01)

Embryogenesis involves the establishment of only the preliminary structure of the apical meristem with roots and the shoot-root axis. The two apical meristems are active after the seed has germinated. The cells are replicated in these meristems, and cell division and differentiation then form the complete plant body. Observations of the dynamics during early seedling development showed that all three kinds of *rgb1* mutants were able to germinate but failed to grow shoots and roots compared with the WT at 1 day after sowing. The shoot-root axis of the WT grew slowly when the seeds were soaked in water for 1 and 3 days and on the first day after sowing (Fig. 5a-c, f-h), but the axis then elongated rapidly on the second day and continued to grow on the third day after sowing (Fig. 5d-e, i-j). The development of the shoot-root axis of the *rgb1–1* and *rgb1–2* mutants was suppressed in 2-day-old and 3-day-old seedlings (Fig. 5l-t, o-w). The length of the shoot-root axis (Fig. 5k) of the *rgb1* mutant was significantly shorter than that of the WT (Fig. 5x). Microscopic observations showed that cell elongation was significantly reduced in the longitudinal plane in the mutant shoot-root axis. However, an obvious increase in cell division was observed in the shoot-root axis of these mutants, possibly due to the cells that had stopped growing. In addition to the limited development of the shoots and roots, the development of the embryo shoot-root axis was suppressed in the *rgb1* mutants.

### Transcriptomic analysis of young rice seedlings from wild-type and *rgb1* mutants

**Fig. 6:**
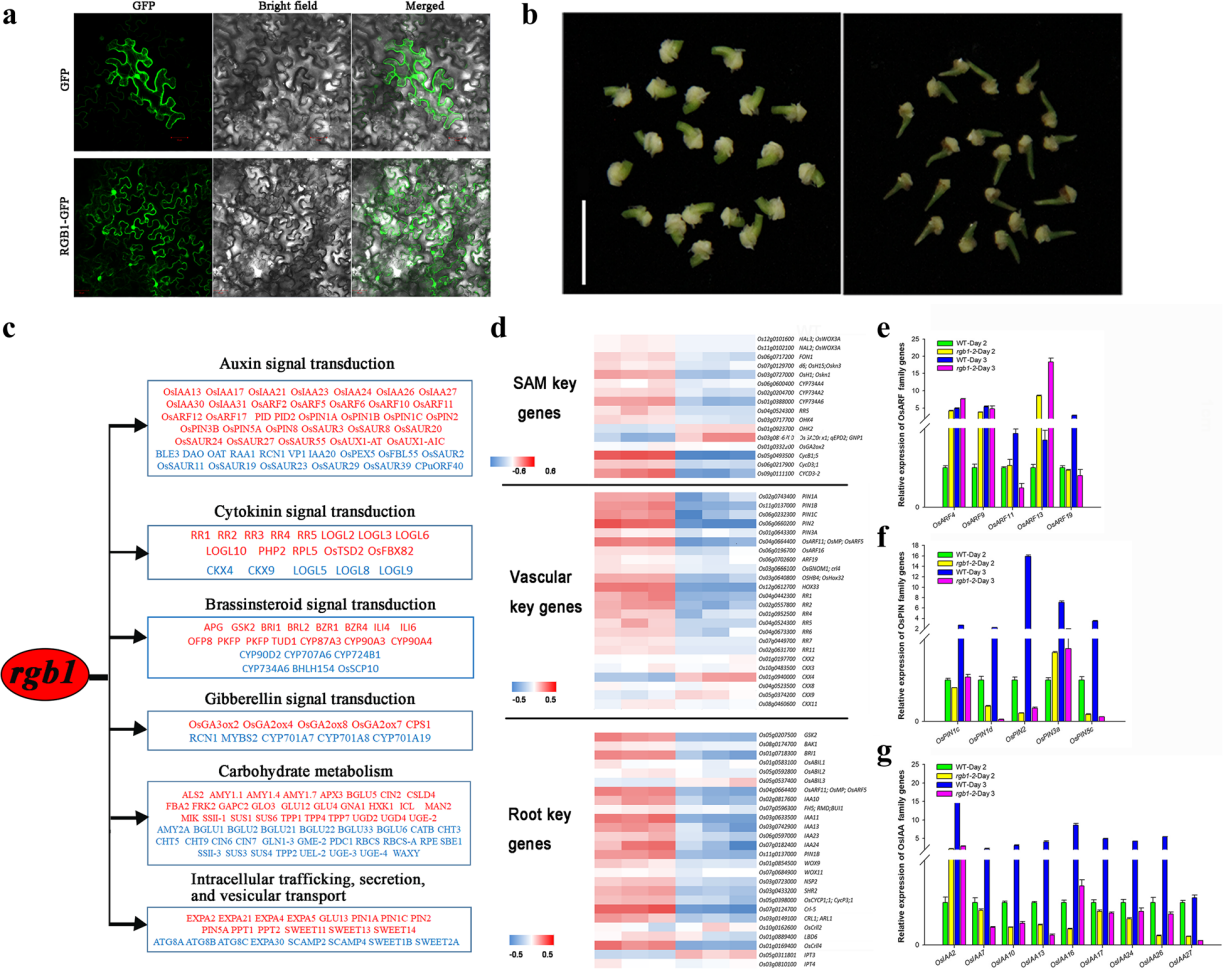
Transcriptomic analysis of young rice seedlings from wild type and *rgb1* mutants. **a** Subcellular localization of RGB1. The localization of GFP alone and RGB1-GFP in *N. benthamiana* epidermal cells are shown. The images were obtained under an optical field to examine cell morphology (bright field), under a dark field to localize green fluorescent protein (GFP), and in combination (merged). M, membrane; N, nucleus. Bars = 50 μm. **b** Tissues of WT and the *rgb1–2* mutant used for RNA-seq and qRT-PCR. **c** Representative genes with known functions (Additional file 11: Table S6, genes with demonstrated functions) in various response/regulatory pathways. The genes whose expression is downregulated or upregulated in the *rgb1–2* mutant and WT are shown in red and blue, respectively. **d** Hierarchical clustering displays of genes specifically expressed in the SAM, vascular tissue, and roots of the *rgb1–2* mutants compared with the WT control. The blue color indicates upregulation, and the red color indicates downregulation in the embryo; the white color indicates that the expression intensity was not detected. **e-g** Transcript levels of auxin signal transduction-related genes at 2 and 3 days after germination. **e** *OsARF* family genes. **f** *OsPIN* family genes. **g** *OsIAA* family genes

In the classic paradigm, G proteins transduce the signal of an activated receptor to downstream effectors, some of which ultimately result in transcriptional control. RGB1-GFP signals were observed to accumulate not only in the plasma membrane but also in the nucleus (Fig. 6a). To investigate the molecular mechanism underlying RGB1, we employed a transcriptome analysis to explore the possible molecular pathway using the embryos of 3-day-old seedlings of the WT and the *rgb1–2* mutant (Fig. 6b). A total of 9,743 differentially expressed genes (DEGs, p-adjust< 0.05 and |log2FC| > = 1) were detected in *rgb1–2* compared with the control, of which 52.54% (5,119 genes) and 47.46% (4,624 genes) were downregulated and upregulated, respectively (Additional file 8: Table S3). To verify the RNA-seq expression data, the auxin signaling genes within the *OsIAA* family were selected for qRT-PCR (quantitative real-time polymerase chain reaction) analysis. Pearson’s correlation test showed a strong correlation (r = 0.85, *p* < 0.05) between the qRT-PCR and RNA-seq data (Additional file 5: Figure S5a). This finding confirmed the reliability of the RNA-seq results obtained in this study. The combination of the seedling developmental characteristics during early postgermination with GO, COG, and KEGG functional enrichment analyses showed that most of the DEGs were enriched in plant hormone signal transduction; carbohydrate metabolism; and intracellular transport, secretion, and vesicular transport (Fig. 6c, Additional file 9: Table S4).

Because RGB1 interferes with seedling growth, we examined the expression of genes specifically expressed in the SAM, the vascular tissues, and the roots in the *rgb1–2* mutant and WT via heatmaps (Fig. 6d, Additional file 10: Table S5). The heatmaps showed that most (81.8%) of the genes were significantly downregulated in the *rgb1*–*2* mutants. In the *rgb1–2* vascular tissues, the auxin transport genes *PIN1A*, *PIN1B*, *PIN1C*, *PIN2*, and *PIN3A*; the auxin-responsive genes *OsARF11*, *OsARF16*, and *OsARF19*; and the cytokinin responsive A-type genes *RR1*, *RR2*, *RR3*, *RR4*, *RR5*, *RR6*, and *RR7* were largely downregulated, while expression of the cytokinin degradation genes *CKX4* and *CKX9* was upregulated. In the roots of *rgb1–2*, the *GSK2*, *BAK1*, and *BRI1* genes in the BR pathway and the auxin-responsive genes *IAA11*, *IAA13*, *IAA23*, and *IAA24* were significantly downregulated. Analysis of the genes listed in Additional file 11: Table S6 for auxin, cytokinin, and brassinosteroids showed that these genes played an important role in seedling development.

The expression of the genes related to auxin signaling and auxin content in 2- and 3-day-old seedlings was examined by qRT-PCR. *OsARF4*, *OsARF9*, and *OsARF13* were upregulated more than 3-fold in the *rgb1* mutants compared to the WT in the 2-day-old seedlings, and *OsARF11* and *OsARF19* were downregulated more than 3-fold compared to the WT in the 3-day-old seedlings (Fig. 6e). In the *rgb1* mutants, *OsPIN1d*, *OsPIN2*, and *OsPIN5C* were downregulated both in 2-day-old and 3-day-old seedlings, and the expression was more than 30-fold greater in the 3-day-old seedlings (Fig. 6f). The expression of *OsIAA2*, *OsIAA7*, *OsIAA10*, *OsIAA13*, *OsIAA16*, *OsIAA17*, *OsIAA24*, *OsIAA26*, and *OsIAA27* was significantly reduced in the *rgb1–2* mutant compared with the WT by > 5-fold at 3 days after germination (Fig. 6g). The relative expression levels of other genes in the *OsARF*, *OsIAA*, and *OsPIN* families are shown in Additional file 5: Figure S5b-d.

## Discussion

CRISPR/Cas9 genome editing technology has been widely used in bacteria, humans, animals, and plants in recent years (Cho et al. 2013; Cong et al. 2013; Jiang et al. 2013; Mali et al. 2013; Yin et al. 2017). The CRISPR/Cas9 system can create small deletions and insertions in specific target genes. Targeted genome editing not only is a valuable tool for basic research but also holds great promise in treating human diseases and in crop improvement and breeding (Gao 2018; Wang et al. 2017). No transgenic plants with greatly decreased expression of *rgb1* were obtained, which hints at the lethal impact of complete *rgb1* mRNAi suppression (Utsunomiya et al. 2012). This result suggests that the complete loss of function of RGB1 cannot be obtained using RNAi technology. We also failed to obtain a homozygous *rgb1* mutant in the T1 and T2 generations when we tested the gene sequence in all the living seedlings; however, we did identify mutant plants that were heterozygous at this locus. It is interesting to determine the stage at which the lethality of *rgb1* occurred. The segregation ratios in the heterozygous lines for the germinated seeds and nongerminated seeds fit a 3:1 ratio, and this result suggests that the germination differences among the *RGB1* heterozygous mutants is controlled by a single gene. The different editing types of the *RGB1* target sequence suggested that we isolated homozygous *rgb1* mutants (Fig. 1b). The identification of homozygous *rgb1* mutants showed that the CRISPR/Cas9 system makes it possible to isolate lethal mutants using only seeds.

In the 1990s, many lethal *Arabidopsis* mutants involved in the development of shoots and roots were isolated and intensively studied (Barton and Poethig 1993). In the past two decades, lethal mutants of rice, such as *osvps22*, *osarid3*, *wox11–1*, and *pps-2* during early postgermination, have been discovered (Nobuhiro et al. 2011; Xu et al. 2015; Zhang et al. 2013; Zhao et al. 2009). However, these mutants have not been studied in depth for their effects on postgermination development. Observations of seedling development during the early postembryonic period showed that the growth of the shoots and roots of the *rgb1* mutants was suppressed and that they stopped developing on the first day after germination (Fig. 2a, d). The induction of callus from embryos of the heterozygous *RGB1/rbg1* mutants showed an independent phenotype. Longitudinal sections of the embryo callus showed that the radicle was brownish black in color, and most of the embryo itself was brown (Fig. 3b, d, e). The brown color of the *rgb1* mutant radicle both under natural growth conditions and on artificial medium for callus induction suggests that the lethality of the *rgb1* mutants and calli was caused by cell death. Extreme dwarfism, early senescence, and seedling lethality are common phenotypes observed in cell death mutants (Lam 2004; Steffens and Sauter 2009; Wu et al. 2018). The phenotype of the *rgb1* mutants was similar to the seedling-lethality phenotype of all *Zmxlg* triple mutant plants and *bak1 bkk1* (BAK1-LIKE 1) double mutants due to spontaneous cell death under sterile growing conditions (Trusov et al. 2007; Wu et al. 2018). Many of the growth and morphological obstacles observed in G protein mutants are attributed to fundamental cellular defects in cell division or elongation. However, the exact molecular mechanisms underlying these phenotypes remain unclear.

Many growth factors activate receptors that transmit signals to the cytoplasm through heterotrimeric G proteins. Several auxin-responsive transporter genes and gene families involved in auxin signaling have been identified, of which the *Aux/IAA*s, *ARF*s, and *PIN*s are the most studied in *Arabidopsis* (Adamowski and Friml 2015; Benjamins and Scheres 2008; Kleine-Vehn and Friml 2008; Leyser 2002; Weijers and Wagner 2016). With respect to our work, it is worth mentioning that the expression of *OsARF11* in the wild type was more than 4-fold greater than that in the *rgb1–2* mutant (Fig. 6e). *PIN1* is expressed in the inner cells of both the shoots and roots, and *PIN2* is expressed in the root epidermis and lateral root cap cells. In rice, *RGB1* is mainly expressed in the root epidermis and vascular cells. The auxin content in the *rgb1* mutant embryos was significantly lower than that in the control embryos at 2 days after germination but was significantly higher than that in the WT embryos at 3 days after germination (Additional file 3: Figure S3b). The low level of expression of *OsARF11*, *OSPIN1d*, *OsPIN2*, and *OsPIN5C* in the *rgb1* mutants (Fig. 6e, f) mainly disrupts the auxin response and transport in vascular tissues and roots and may suppress elongation of the root cells. GAs and BRs are important for nutrient metabolism and transport. Active GAs can promote the secretion of amylase, which converts starch into glucose (Gubler et al. 1995). BRs regulate components of other hormone signaling pathways, seed germination, and photomorphogenesis in the dark (Szekeres et al. 1996). The GA3 and BL contents in the *rgb1* mutant were much higher than that in the control in the embryos of 3-day-old seedlings (Additional file 3: Figure S3c, f). The loss of function of *RGB1* results in large transcript level changes and unbalanced endogenous hormone contents that suppress seedling formation in rice. However, the exact molecular mechanism of the lethality of the *rgb1* mutants requires additional detailed evidence. The obtained *rgb1* mutants constitute an ideal material for exploring the molecular mechanism underlying G proteins in rice.

In this study, we show that seeds of the *rgb1* mutants could germinate but failed to form seedlings under natural conditions. Following germination, the storage compounds of the starchy endosperm are hydrolyzed by the scutellum epithelial cells (Domínguez and Cejudo 2014). The nutrients in the scutellum flow into the vascular bundle of the embryo and are then transferred to promote the growth of the shoots and roots in rice. At the same time, developing seeds are under oxidative stress to promote the embryo to take up nutrients. In the *rgb1* mutants, the starch around the embryo was consumed, but the development of the shoots and roots was suppressed (Fig. 4d). A fully functional vascular system is needed immediately after seed germination. A recurring feature is the expression of *RGB1* in the vascular tissue of the embryo during the entire early postembryonic developmental period (Fig. 2h, i, j). This indicates that RGB1 plays an important role in nutrient transport. Another recurring feature is the expression of *RGB1* in the xylem and the epidermis of roots in the WT. The *RGB1* expression pattern is similar to that of *AGB1* in *Arabidopsis*; however, the morphology of the *agb1* mutants did not significantly change under natural conditions. The difference in nutrient storage organs and transport patterns between monocotyledons and dicotyledons may be responsible for the functional differentiation of Gβ.

## Conclusions

**Fig. 7:**
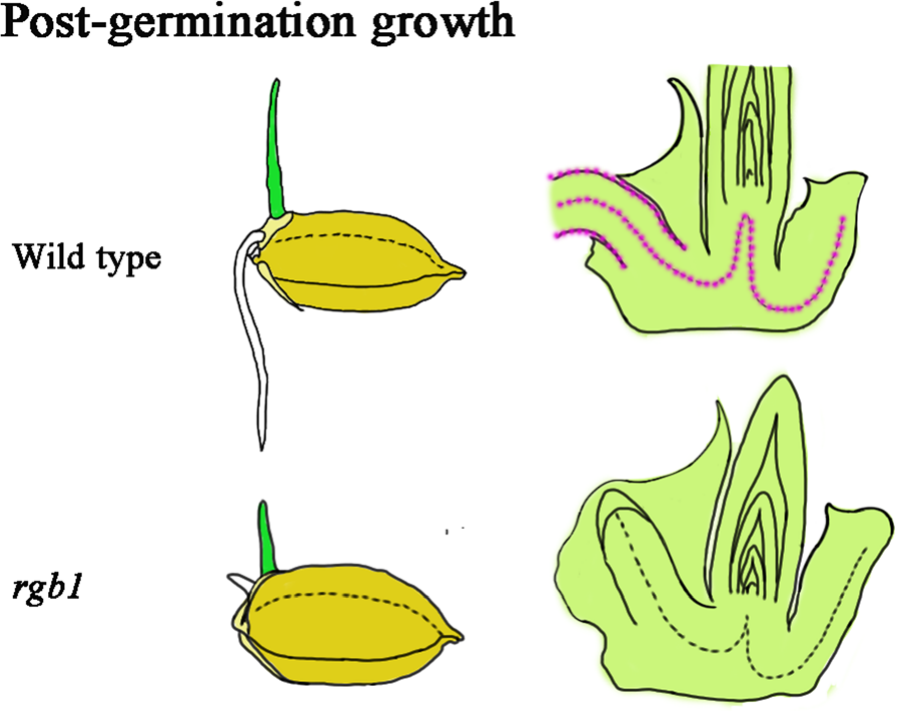
Model of RGB1 function in early postgermination development in rice. *RGB1* specifically functions in vascular tissue and epidermal cells (pink) in rice embryos during the early postembryogenic development stage. The development of the shoots, roots, and the embryo shoot-root axis is significantly suppressed in the *rgb1* mutants and the *rgb1* mutants are early postgermination lethal

In conclusion, using only seeds, we have demonstrated a method to identify lethal mutants in rice, and we applied this method to identify lethal *rgb1* mutants. The results showed that *RGB1* is expressed specifically in vascular tissues in rice embryos during early postembryogenic development and that *RGB1* loss-of-function mutants stop growing on the first day after germination because of cell death. Overall, our results suggest that *RGB1* is indispensable for seedling formation but not for germination (Fig. 7).

## Methods

### Plant materials and plant growth conditions

The *japonica* rice varieties ‘Nipponbare’ and ‘Zhonghua 11’ were used for genetic transformation in this study. Mature seeds were harvested from T0-, T1-, and T2-generation transgenic plants and subsequently dried for 36–48 h at 45 °C to break dormancy for the assays.

For IAA, BR, GA treatment, the germinated seeds of WT and the *RGB1/rgb1* mutant lines were grown on filter paper atop wet sand for 1 week. The sand was immersed in water containing 1 μM or 5 μM IAA, 1 μM or 3 μM BRs, or 1 μM GA.

For the auxin treatment, 7-day-old seedlings were treated with mock or 10 μM NAA for 0.25 h, 0.5 h, 1 h, 4 h, and 7 h, after which the shoots and roots were collected for qRT-PCR (quantitative real-time RT-PCR) analysis.

### Vector construction and rice transformation

We designed a CRISPR/Cas9 gene editing construct in which the sgRNA target was located within the first WD40 domain of the *RGB1* gene to inactivate the RGB1 protein. A *p35S-RGB1_Cas9* vector construct was introduced into embryogenic calli of the *japonica* rice cultivar ‘Zhonghua 11’ (ZH11) via Agrobacterium-mediated transformation. The fragment adding the linker was cloned into a BGK01 vector, after which the plasmid was transformed into ‘Zhonghua 11’ (Biogle company).

To fuse the *RGB1* gene promoter and the GUS coding sequence (RGB1::GUS), the 2.137-kb (with 105 from the ATG) RGB1 promoter fragment was amplified from ‘Nipponbare’ genomic DNA and inserted into a pCAMBIA1301 plasmid between the BamHI and NcoI sites. The primers used in PCR amplification were RGB1_GUS_1F and RGB1_GUS_1R. The plasmid was subsequently transformed into ‘Nipponbare’, generating the RGB1::GUS transformed plants.

### Genomic DNA extraction and mutant screening

Dehulled heterozygous seeds, heterozygous mature seeds, and seeds of WT were used for germination tests. The dehulled rice seeds were sterilized with 70% ethyl alcohol and soaked in 1 N HNO_3_ for 24 h. Forty germinated seeds were then sown on a double layer of filter paper on top of wet silica sand in square transparent plastic petri dishes (10 cm × 10 cm) (excess water was removed) under conditions of 14 h light/10 h of darkness and 30/25 °C. The seedlings were counted on the 7th day after germination. In another germination assay, normal seeds were soaked in distilled water for 3 days, and 96 of the germinated seeds were sown in a PCR plate in which the bottom 3 mm of the wells had been removed. The PCR plate containing the germinated seeds was then placed in a box containing 1 l of distilled water, and the seedlings were allowed to grow under the same conditions as those above for 5 days before they were counted (the shoot ends were above the water level). Of the 5-day-old seedlings, those with defects in their shoots and roots were isolated.

Genomic DNA extraction from the leaves of T0-, T1-, and T2-generation transgenic seedlings was performed using the traditional CTAB method. Genomic DNA from individual hulled seeds whose seed coats were removed was extracted as follows: the dehulled seeds were ground in 2 ml microcentrifuge tubes, after which 100 μl of distilled water was added. The tissue was solubilized by adding 500 μl of CTAB extraction buffer (2% w/v CTAB, 1.4 mol/L NaCl, 0.1 mol/L Tris-base, 20 mmol/L EDTA, pH = 8) to the tubes, which were incubated at 65 °C for 30 min. We then added 350 μl of trichloromethane with 350 μl of DNA extraction phenol reagent (trichloromethane:DNA extraction phenol reagent = 1:1), after which the tubes were centrifuged at 13,400 x g for 10 min. The aqueous layers (500 μl) were transferred to new 2.0 ml tubes, and 250 μl of trichloromethane together with 250 μl of DNA extraction phenol reagent was added to each tube. The tubes were then mixed and centrifuged as before, and 500 μl of the aqueous layers was transferred to 1.5 ml tubes. Afterward, 500 μl of isopropyl alcohol was added to each tube, and the tubes were subsequently incubated at − 20 °C for 30 min. The nucleic acids were recovered by centrifugation for 10 min at 13,400 x g. The liquid was discarded, and the pellets were washed in 70% ethanol for 5 min. The tubes were incubated at 37 °C for 3 h to dry the pellets (they could also have been incubated at room temperature overnight), after which the pellets were dissolved in 200 μl of ddH_2_O.

DNA fragments containing the RGB1 Cas9 target sequence were amplified using the primers RGB1_Check_1F and RGB1_Check_1R from DNA isolated from both leaves and individual seeds without coats or embryos and then sequenced. The *rgb1* mutants without Cas9 proteins were isolated from the transgenic plants using the primers PUV3_R_F and using gRNA_R5_R, HYG_F and HYG_R.

### Pollen viability assays

Evaluation of pollen grain viability of the *RGB1/rgb1* heterozygous mutants was performed as previously described (Xiao et al. 2015). Anthers from mature rice spikelets were crushed with tweezers in distilled water on a glass slide and then stained with 1% I_2_-KI solution for 5 min. The samples were subsequently observed and imaged with a light microscope (DM 1000, Leica). The frequency of darkly stained pollen grains was determined for at least five plants of each line.

### Microscopic observations

Embryos (at 4 days after germination) of fresh young seedlings were dissected longitudinally and observed using a dissecting microscope (Olympus). The embryos at different stages were fixed in FAA solution (50% ethanol, 5% acetic acid, 2.4% formaldehyde) for more than 24 h, dehydrated, embedded in paraffin, and then sectioned. The sections (7 μm thick) were then transferred to a slide and examined using a light microscope (DM 1000, Leica). Cell lengths within the primary root were measured using Adobe Photoshop CS2 software. For primary root cell observations, differentiation was observed using a light microscope (DM 1000, Leica).

### RNA extraction and qRT-PCR assays

Total RNA was isolated using an RNA extraction kit (Tian, China). A FastQuant RT Kit was used to synthesize first-strand cDNA in accordance with the manufacturer’s instructions (Tiangen, China). The full-length or truncated cDNAs of RGB1 from ‘Nipponbare’ were amplified and sequenced, and the rice housekeeping gene Actin (LOC_Os11g06390) was used as an internal control for the normalization of gene expression. Real-time PCR analysis was performed on an Applied Biosystems ViiA7 Real Time PCR System using the SYBR Green method, and the primer sequences used are given in Additional file 6: Table S1.

### Callus induction assays

Seeds of heterozygous *RGB1* mutants were cultured on callus induction medium (4.43 g/L MS medium (PhytoTechnology Laboratories), 0.5 g of casamino acid, 30 g of sucrose, 2.5 g of Phytagel; the pH was adjusted to 5.8 (NaOH)) containing 2,4-D (3.6 mg/L) and 6-BA (1 mg/L); 40 seeds were sown per sterile petri dish. The dishes were wrapped with Parafilm (Sigma-Aldrich) and then incubated in the dark at 28 °C. The seeds were observed for callus growth and microbial contamination for a period of 10 days. The length of the shoots was measured, and the tips of the embryos were cut for microscopic examination and then imaged.

### Promoter-GUS fusion studies

The developing transgenic plants at different early postembryogenic developmental stages were incubated in substrate buffer (CAT. RTU4032, Real Times) for 20–22 h at 37 °C. After they were stained, the seedlings were washed with 70% ethanol to remove the chlorophyll. GUS staining of the longitudinally dissected embryos was examined with a dissecting microscope (Olympus MVX10).

### Subcellular localization of RGB1 proteins

For subcellular localization analysis, the coding sequence of RGB1 was fused in frame to the GFP coding sequence in a pCAMBIA1300-221GFP vector to generate RGB1-GFP. GFP alone was used as a control. Both sequences were driven by the CaMV 35S promoter. These two fusion constructs were introduced into *A. tumefaciens* strain EHA105. A tobacco (*Nicotiana benthamiana*) leaf was infiltrated with the transformed *A. tumefaciens* to express the GFP fusion protein. Approximately 48 h later, the tobacco epidermal cells were observed and imaged using a confocal microscope (LSM710, Zeiss).

### In situ hybridization

Embryos from fresh 1-day-old, 3-day-old, and 4-day-old seedlings were used for in situ hybridization. Paraffin sections from different tissues during early postembryonic development were obtained. An *RGB1* probe was amplified using the gene-specific primers In-RGB1-F and In-RGB1-R. The PCR fragments were then inserted between the SpeI and SalI sites of a pGEM-T plasmid (Promega) and transcribed in vitro using a digoxigenin RNA labeling kit (Roche).

### TUNEL reaction

An In Situ Cell Death Detection Kit (TUNEL, Cat. No. 11 684 817 910, Sigma-Aldrich) was used as directed with embryo sections from 1-day-old wild type and 4-day-old *rgb1* mutant seeds, with slight modifications. Sections were washed twice with xylene for 5 min each and then washed with an ethanol series (absolute, 95%, 90%, 80%, 70%, diluted in double-distilled water) for 3 min/time. The tissue sections were subsequently incubated for 15–30 min at 21 to 37 °C with proteinase K working solution and then washed twice with PBS. For the preparation of the TUNEL reaction mixture, fixed and permeabilized cells were incubated in TdT (50 μl/well) and 450 μl of dUTP; the negative control contained only 50 μl of dUTP, and the positive control was treated first with 100 μl of DNase I for 10 min, after which the fixed and permeabilized cells were subsequently incubated in TdT (50 μl/well) and 450 μl of dUTP. The area around the samples was dried, and 50 μl of TUNEL reaction mixture was added to the samples, which were then covered and incubated for 60 min at 37 °C in a humid atmosphere in the dark. The slides were subsequently rinsed three times with PBS. The samples were then analyzed in a drop of PBS under a fluorescence microscope with an excitation wavelength in the range of 450–500 nm and with a detection wavelength in the range of 515–565 nm (green).

### RNA sequencing analysis

The embryos of ‘Zhonghua11’ and the *rgb1–2* mutant at 3 days after germination were harvested for total RNA extraction using TRIzol reagent (Invitrogen), and the samples were further purified using an mRNA purification kit (Invitrogen). All samples of total RNA were assessed based on the following criteria: OD 260/A280 ≈ 2.1, OD 260/230 ≈ 2.0, quantity> 15 μg, and good physical integrity. Three biological replicates were used for RNA sequencing of each sample. The RNA-seq libraries were sequenced on an Illumina HiSeq 2000 instrument (100-nt paired-end reads) by Majorbio, Inc. (Shanghai, China). The filtered clean reads were aligned to the rice ‘Nipponbare’ reference genome and genes (http://rice.plantbiology.msu.edu/) using TopHat2 and Bowtie2 software. The data were analyzed using the free online Majorbio I-Sanger Cloud platform (http://www.i-sanger.com).

## Additional files

Additional file 1: **Figure S1.** Pollen viability and embryo structures in the *RGB1/rgb1* heterozygous mutant lines. **(a)** Pollen viability in the WT and *RGB1/rgb1* heterozygous mutants (Bar = 200 μm). The pollen grains were stained with 1% I_2_–KI and imaged with a Leica DM 1000 light microscope. **(b)** Median longitudinal sections of WT and *RGB1/rgb1* heterozygous mutant seeds (18 days after flowering); a plumule, b radicle. (DOCX 3952 kb)

Additional file 2: **Figure S2.** Phenotypes and genotypes of the *rgb1* mutants; relative expression of G protein genes in the embryos of the WT and *rgb1* mutants. **(a-d)** DNA sequences and phenotypes of the WT and *rgb1* mutants. **(a)** WT, **(b)** *rgb1–1*, **(c)** *rgb1–2*, **(d)** *rgb1–3*. Mutations are shown in red. **(e)** Relative expression of *RGB1* in the embryos of the WT and the *rgb1–1* and *rgb1–2* mutant lines, as determined by qRT-PCR. The forward primer used in the qRT-PCR assays was the RGB1-Cas9 target sequence. **(f-h)** Relative expression of G protein family genes in the embryos of the WT and the *rgb1–1* and *rgb1–2* mutant lines from **(f)** 1-day-old seedlings, **(g)** 2-day-old seedlings, and **(h)** 3-day-old seedlings. (DOCX 2619 kb)

Additional file 3: **Figure S3.** Induction of *RGB1* expression by NAA and measurements of the concentrations of endogenous hormones in the *rgb1* mutant and WT. **(a)** Relative expression of *RGB1* in 7-day-old wild-type seedlings grown in mock medium and in medium supplemented with 10 μM NAA. **(b-f)** Mass spectrometric measurements of different endogenous hormones in the embryos of 3-day-old seedlings. **(b)** IAA, **(c)** BRs, **(d)** IPA, **(e)** ABA, **(f)** GA_3_. (DOCX 2663 kb)

Additional file 4: **Figure S4.** Stages of seed germination and early postgermination seedlings of rice. (DOCX 4775 kb)

Additional file 5: **Figure S5.** Comparison of gene expression determined by qRT-PCR and RNA-seq and relative expression of auxin signaling-related genes in the embryos of the WT and *rgb1–2* mutants at 2 and 3 days after germination. **(a)** Comparison of relative gene expression levels determined by RNA-seq and qRT-PCR. Pearson’s test indicated a strong correlation between the two techniques (r = 0.85; *p* < 0.05). **(b-d)** Relative expression of auxin-related family genes. Gene expression was determined in the WT and *rgb1–2* mutant embryos at 2 and 3 days after germination. **(b)** *OsIAA*; **(c)** *OsARF*; **(d)** *OsPIN (DOCX 476 kb)*

Additional file 6: **Table S1.** Oligonucleotide primers and probe sequences used. (DOC 113 kb)

Additional file 7: **Table S2.** Genotypes of the seeds in the T0-, T1-, and T2-generation transgenic seedlings. (XLSX 16 kb)

Additional file 8: **Table S3.** Number of differentially expressed genes identified in the transcriptome analysis. (DOCX 13 kb)

Additional file 9: **Table S4.** GO, COG, and KEGG classification tables. (XLSX 77 kb)

Additional file 10: **Table S5.** Genes specifically expressed in the SAM, vascular tissues and developing roots. (XLSX 13 kb)

Additional file 11: **Table S6.** Gene expression involved in various response/regulatory pathways. (XLSX 82 kb)

## Data Availability

All the data generated or analyzed during this study are included in this published article and its additional files.

## Abbreviations

2,4-D: 2,4-dichlorophenoxyacetic acid
6-BA: 6-benzylaminopurine
BL: Brassinolide
BR: Brassinosteroid
COG: Clusters of orthologous groups of proteins
CRISPR/Cas9: Clustered regularly interspaced short palindromic repeats and CRISPR-Associated protein 9
CTAB: Hexadecyl trimethyl ammonium bromide
DEGs: Differentially expressed genes
GA: Gibberellin
GFP: Green fluorescent protein
GO: Gene ontology
GUS: β-glucuronidase
IAA: Indole-3-acetic acid
IPA: Isopentenyl adenosine
KEGG: Kyoto encyclopedia of genes and genomes
NAA: 1-Naphthylacetic acid
PCD: Programmed cell death
qRT-PCR: Quantitative real-time polymerase chain reaction
RNA-seq: RNA sequencing
SAM: Shoot apical meristem
TUNEL: TdT-mediated dUTP nick-end labeling
ZH11: Zhonghua 11
