# Supplementary material for: The heterotrimeric G protein β subunit RGB1 is required for seedling formation in rice
Source: Rice (N Y). 2019 Jul 18;12:53. doi: 10.1186/s12284-019-0313-y (PMC6639528; doi:10.1186/s12284-019-0313-y)
Supplement: Supplementary file 1 — Figure S1. Pollen viability and embryo structures in the RGB1/rgb1 heterozygous mutant lines. (a) Pollen viability in the WT and RGB1/rgb1 heterozygous mutants (Bar = 200 μm). The pollen grains were stained with 1% I2–KI and imaged with a Leica DM 1000 light microscope. (b) Median longitudinal sections of WT and RGB1/rgb1 heterozygous mutant seeds (18 days after flowering); a plumule, b radicle. (DOCX 3952 kb) [file 12284_2019_313_MOESM1_ESM.docx]

**
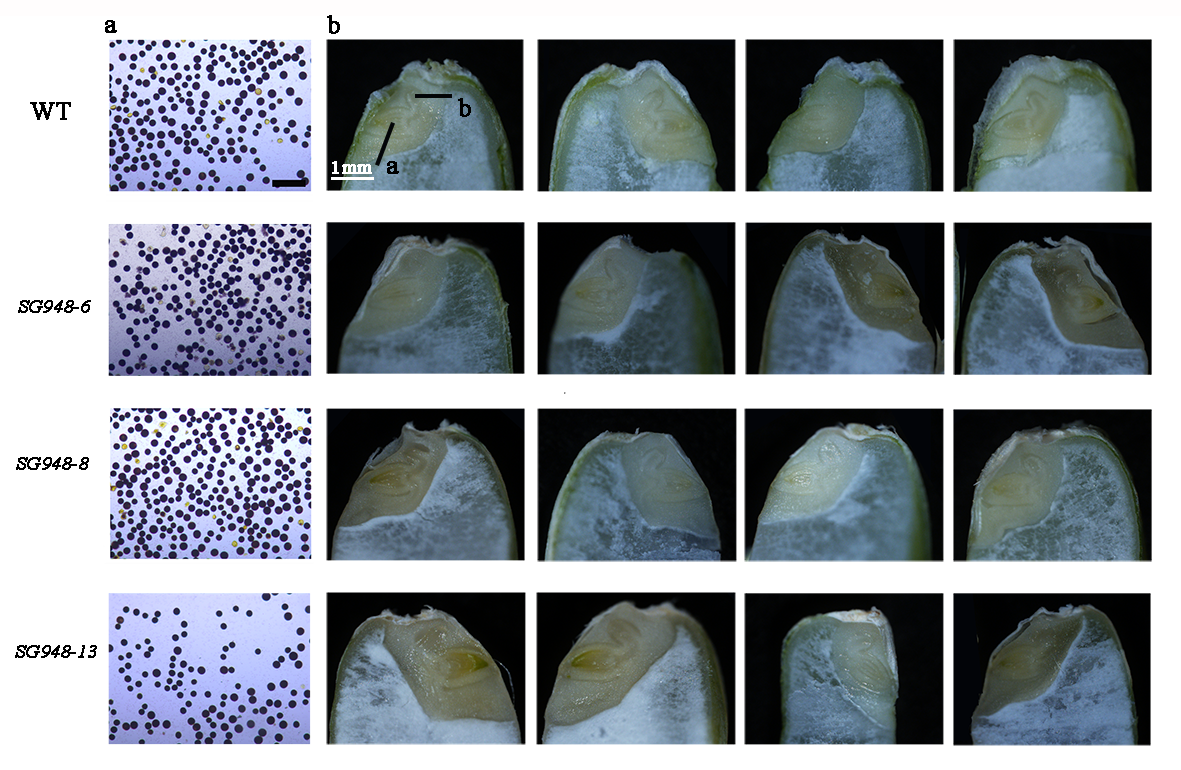
**

**Figure S1.** Pollen viability and embryo structures in the *RGB1/rgb1* heterozygous mutant lines. **(a)** Pollen viability in the WT and *RGB1/rgb1* heterozygous mutants (Bar=200 µm). The pollen grains were stained with 1% I_2_–KI and imaged with a Leica DM 1000 light microscope. **(b)** Median longitudinal sections of WT and *RGB1/rgb1* heterozygous mutant seeds (18 days after flowering); a plumule, b radicle.
