# Supplementary material for: The heterotrimeric G protein β subunit RGB1 is required for seedling formation in rice
Source: Rice (N Y). 2019 Jul 18;12:53. doi: 10.1186/s12284-019-0313-y (PMC6639528; doi:10.1186/s12284-019-0313-y)
Supplement: Supplementary file 2 — Figure S2. Phenotypes and genotypes of the rgb1 mutants; relative expression of G protein genes in the embryos of the WT and rgb1 mutants. (a-d) DNA sequences and phenotypes of the WT and rgb1 mutants. (a) WT, (b) rgb1–1, (c) rgb1–2, (d) rgb1–3. Mutations are shown in red. (e) Relative expression of RGB1 in the embryos of the WT and the rgb1–1 and rgb1–2 mutant lines, as determined by qRT-PCR. The forward primer used in the qRT-PCR assays was the RGB1-Cas9 target sequence. (f-h) Relative expression of G protein family genes in the embryos of the WT and the rgb1–1 and rgb1–2 mutant lines from (f) 1-day-old seedlings, (g) 2-day-old seedlings, and (h) 3-day-old seedlings. (DOCX 2619 kb) [file 12284_2019_313_MOESM2_ESM.docx]

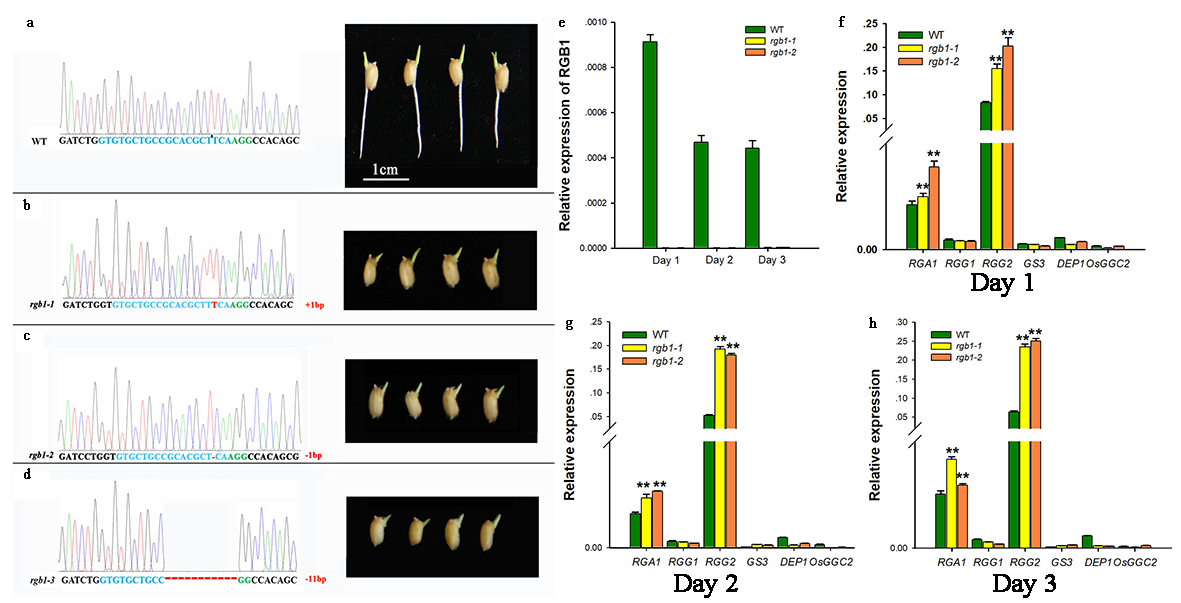


**Figure S2.** Phenotypes and genotypes of the *rgb1* mutants; relative expression of G protein genes in the embryos of the WT and *rgb1* mutants. **(a-d)** DNA sequences and phenotypes of the WT and *rgb1* mutants. **(a)** WT, **(b)** *rgb1-1*, **(c)** *rgb1-2*, **(d)** *rgb1-3*. Mutations are shown in red. **(e)** Relative expression of *RGB1* in the embryos of the WT and the *rgb1-1* and *rgb1-2* mutant lines, as determined by qRT-PCR. The forward primer used in the qRT-PCR assays was the RGB1-Cas9 target sequence. **(f-h)** Relative expression of G protein family genes in the embryos of the WT and the *rgb1-1* and *rgb1-2* mutant lines from **(f)** 1-day-old seedlings, **(g)** 2-day-old seedlings, and **(h)** 3-day-old seedlings.
