# Supplementary material for: The heterotrimeric G protein β subunit RGB1 is required for seedling formation in rice
Source: Rice (N Y). 2019 Jul 18;12:53. doi: 10.1186/s12284-019-0313-y (PMC6639528; doi:10.1186/s12284-019-0313-y)
Supplement: Supplementary file 3 — Figure S3. Induction of RGB1 expression by NAA and measurements of the concentrations of endogenous hormones in the rgb1 mutant and WT. (a) Relative expression of RGB1 in 7-day-old wild-type seedlings grown in mock medium and in medium supplemented with 10 μM NAA. (b-f) Mass spectrometric measurements of different endogenous hormones in the embryos of 3-day-old seedlings. (b) IAA, (c) BRs, (d) IPA, (e) ABA, (f) GA3. (DOCX 2663 kb) [file 12284_2019_313_MOESM3_ESM.docx]

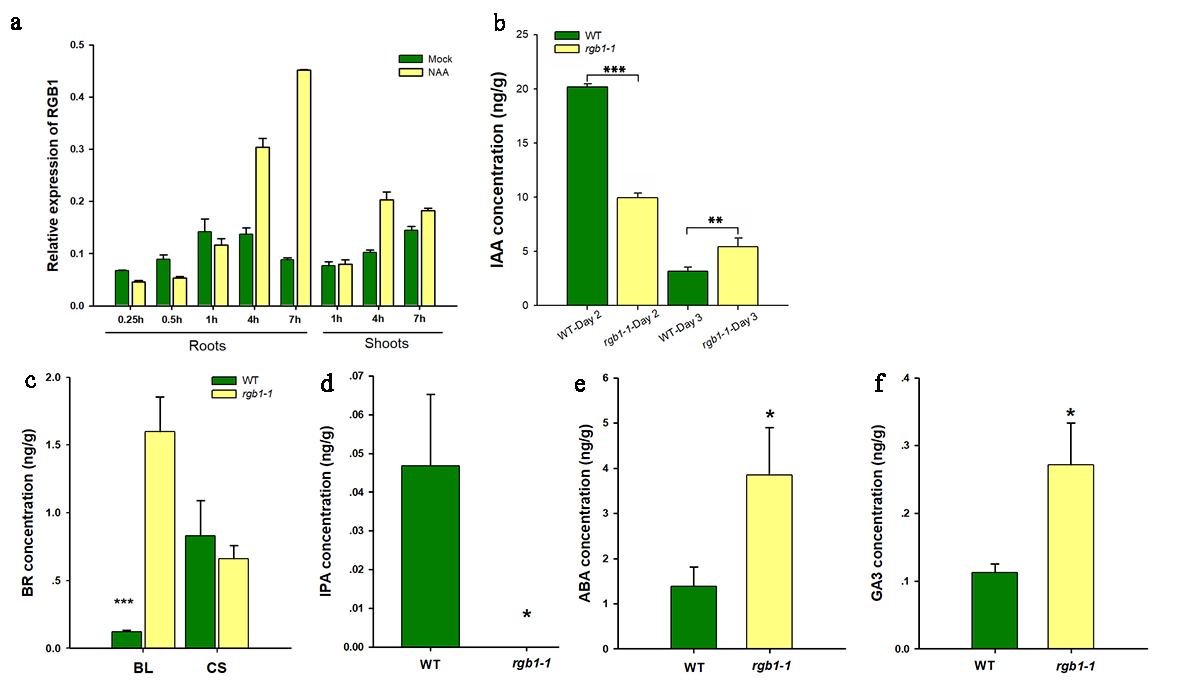


**Figure S3.** Induction of *RGB1* expression by NAA and measurements of the concentrations of endogenous hormones in the *rgb1* mutant and WT. **(a)** Relative expression of *RGB1* in 7-day-old wild-type seedlings grown in mock medium and in medium supplemented with 10 Μm NAA. **(b-f)** Mass spectrometric measurements of different endogenous hormones in the embryos of 3-day-old seedlings. **(b)** IAA, **(c)** BRs, **(d)** IPA, **(e)** ABA, **(f)** GA_3_.
