# Supplementary figures and images for: The heterotrimeric G protein β subunit RGB1 is required for seedling formation in rice
Source: Rice (N Y). 2019 Jul 18;12:53. doi: 10.1186/s12284-019-0313-y (PMC6639528; doi:10.1186/s12284-019-0313-y)

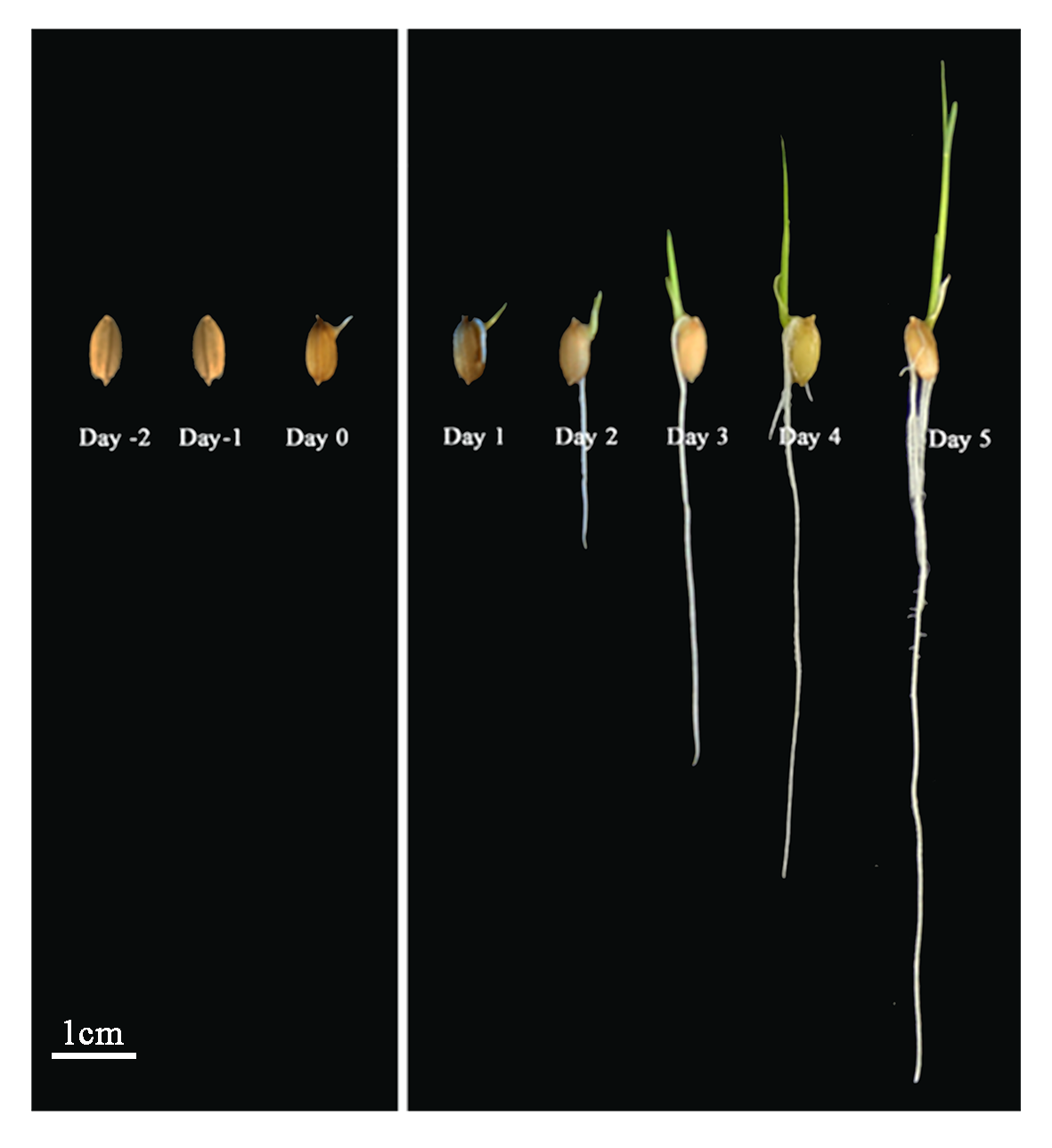


**Figure S4.** Stages of seed germination and early postgermination seedlings of rice.

Supplement: Supplementary file 4 — Figure S4. Stages of seed germination and early postgermination seedlings of rice. (DOCX 4775 kb) [file 12284_2019_313_MOESM4_ESM.docx]
