# Supplementary material for: The heterotrimeric G protein β subunit RGB1 is required for seedling formation in rice
Source: Rice (N Y). 2019 Jul 18;12:53. doi: 10.1186/s12284-019-0313-y (PMC6639528; doi:10.1186/s12284-019-0313-y)
Supplement: Supplementary file 5 — Figure S5. Comparison of gene expression determined by qRT-PCR and RNA-seq and relative expression of auxin signaling-related genes in the embryos of the WT and rgb1–2 mutants at 2 and 3 days after germination. (a) Comparison of relative gene expression levels determined by RNA-seq and qRT-PCR. Pearson’s test indicated a strong correlation between the two techniques (r = 0.85; p < 0.05). (b-d) Relative expression of auxin-related family genes. Gene expression was determined in the WT and rgb1–2 mutant embryos at 2 and 3 days after germination. (b) OsIAA; (c) OsARF; (d) OsPIN (DOCX 476 kb) [file 12284_2019_313_MOESM5_ESM.docx]

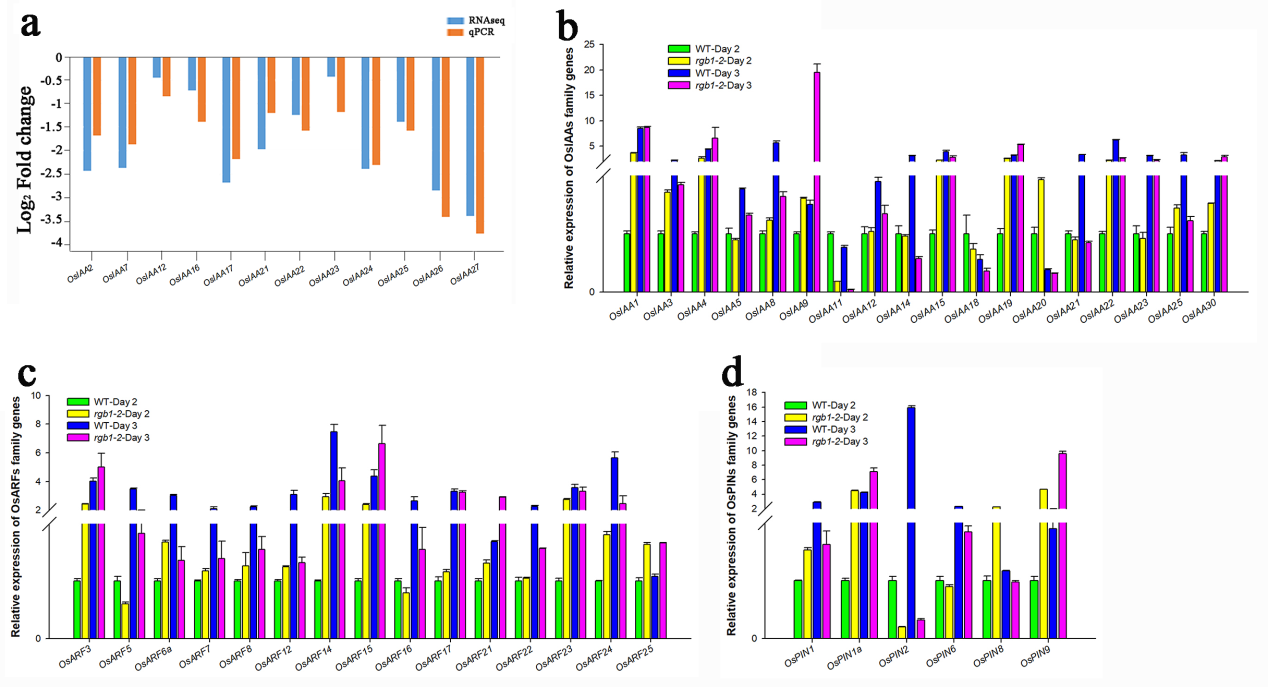


**Figure S5.** Comparison of gene expression determined by qRT-PCR and RNA-seq and relative expression of auxin signaling-related genes in the embryos of the WT and *rgb1-1* mutants at 2 and 3 days after germination. **(a)** Comparison of relative gene expression levels determined by RNA-seq and qRT-PCR. Pearson’s test indicated a strong correlation between the two techniques (r = 0.85; p<0.05). **(b-d)** Relative expression of auxin-related family genes. Gene expression was determined in the WT and *rgb1-2* mutant embryos at 2 and 3 days after germination. **(b)** *OsIAA*; **(c)** *OsARF*; **(d)** *OsPIN*
