# Supplementary material for: The heterotrimeric G protein β subunit RGB1 is required for seedling formation in rice
Source: Rice (N Y). 2019 Jul 18;12:53. doi: 10.1186/s12284-019-0313-y (PMC6639528; doi:10.1186/s12284-019-0313-y)
Supplement: Supplementary file 6 — Table S1. Oligonucleotide primers and probe sequences used. (DOC 113 kb) [file 12284_2019_313_MOESM6_ESM.doc]

Table S1 Primer sequences for qRT–PCR analysis and vector generation and probe sequences for *In situ* hybridization

| **Primers for qRT-PCR** | | | | | |
| --- | --- | --- | --- | --- | --- |
| Gene name | Sequences (5’ -3’) | | | | Experiments |
| *OsARF3* | CAAGCACACATGGAGGGTT / CGAGGTTGACCTCTATAAATATG | | | | qRT-PCR |
| *OsARF4* | GAGTCTTGAGAGAGAGATGC / GCATCAGCCATCAGATAAGC | | | | qRT-PCR |
| *OsARF5-1* | CACTCACCGCAAGTGATACA/ CTGGAGGTTGCATAGAGAAGTC | | | | qRT-PCR |
| *OsARF5-2* | GTCCCTTGGACAGTTGGTAAA/ TGGCTGTGAGAGTGTTGATG | | | | qRT-PCR |
| *OsARF6-1* | CATGAACTTCGTAGTGAGGTAGG/ CGTCCTCTCGGTCAACAAATA | | | | qRT-PCR |
| *OsARF6-2* | AGGCTGGATAGTTCCCTACTT/ CAGCATGGCAGGTGATACTT | | | | qRT-PCR |
| *OsARF7* | GTTATCCGCTGGTTGGTTC/ CCAATCACGCAGGGAATG | | | | qRT-PCR |
| *OsARF8-1* | CCATCAACCTCTCGTCTTTCTC/ CGGGTGGTTGTGATGGTAAT | | | | qRT-PCR |
| *OsARF8-1* | GAATCAGCACTGACCTGACA / TCTGCCCGAACAGCATTAG | | | | qRT-PCR |
| *OsARF9* | CTGTGCTGAAGACGCAAC/ ACCAGACAAGAACGCCAAG | | | | qRT-PCR |
| *OsARF11-1* | CTGAAGAGAGTGCAAGTCAGAG/ CCCAGTAGACATTGGTTGAGAG | | | | qRT-PCR |
| *OsARF11-2* | AACTACCCAAACCTGCCATC/ CTGAAGGGTCATCTGTGCATAA | | | | qRT-PCR |
| *OsARF12* | CCCAGGAGCTAATTGCAC/ CTGGCACGTCTTATTCCAAG | | | | qRT-PCR |
| *OsARF13-1* | TCAATGGCACCCTCACTAAC/ TGGACCTGCCAAGGATTAAC | | | | qRT-PCR |
| *OsARF13-2* | CCTGTTGATTTCCCTCCTCTTC/ GGTTGCGGGTGCATTAGTAT | | | | qRT-PCR |
| *OsARF14* | GTCGTTGCATCAAGCTACCT/ TGCATCATGTGGCACCAAC | | | | qRT-PCR |
| *OsARF15* | CTGGAGGTGAAGTAACACTAG/ CAGAATCATCAGTCCCTCC | | | | qRT-PCR |
| *OsARF16* | CTCGTGCCGCCTACTTTAG/ CCTCCGGATACACGAATATC | | | | qRT-PCR |
| *OsARF17-1* | GGGTAGCCGAGTGGTTTATTT/ TAAGTTGTGGAGGCAAGTTAGG | | | | qRT-PCR |
| *OsARF17-2* | CCGGAAGCTGTAGAAGAACAA/ CTGTGACCCTGTGGGAAATAA | | | | qRT-PCR |
| *OsARF19* | GCAGTTATCAGAGCTTGCAC/ TGCAGGTGGTTGCATTGAG | | | | qRT-PCR |
| *OsARF20-1* | CCCGATCTCCTACAGGATACA/ ATTCGCACAGCCGAAGTAG | | | | qRT-PCR |
| *OsARF20-2* | ACGCCATGATTTCCCTCTTT/ TCTTGTCGAAGAAGCAGAACTC | | | | qRT-PCR |
| *OsARF21* | GAGCCTATCATTGCTCCATTCT/ CTCTTGAGAAGACCGTCCATTT | | | | qRT-PCR |
| *OsARF22* | AGGCTTCTGACCTTGGTTTAG/ CAGCCGACCATACAGTTCTT | | | | qRT-PCR |
| *OsARF23* | ACAAGAAGATAACGGCTCTAC/ ATGTGTGCTCGTGTCCGAG | | | | qRT-PCR |
| *OsARF24* | TGGTGGCCAAGGATCTTC/ CGTAGTTCCCCATTCTCAC | | | | qRT-PCR |
| *OsARF25* | ATGAAGGGGAGGATTTGCTG/ GAAGCTGCTGGTCAGCTTC | | | | qRT-PCR |
| *OsIAA1* | GATGGACATCTCTGGCCTTATAC/ GCACATCATAGAGCACAGAACTA | | | | qRT-PCR |
| *OsIAA2* | GGACTAGTAGGGAGTAGTGATTCT/ GGCCGTCAGGTACTATTCAAA | | | | qRT-PCR |
| *OsIAA3* | GATGGCAACCAACCAGATAAAG/ AGGTCCACCTTCCTCAGATA | | | | qRT-PCR |
| *OsIAA4* | TGTTCGTGAAGGTGTACATGG/ ATGTGGCAGAGCTTGATGAG | | | | qRT-PCR |
| *OsIAA6* | GAGATTACAGGCTACAGCAAGAG/ TTTGCTCCAGTTGTCGTAGTAG | | | | qRT-PCR |
| *OsIAA7* | GCAAGGCAAGGCAAACAA/ TTGTCTGGAAGCAGCCATTA | | | | qRT-PCR |
| *OsIAA8* | CAGAGCATCCATCACCTATCATC/ CCTGGTCTTCGTATGTCAGAAC | | | | qRT-PCR |
| *OsIAA9* | GATTGCAGGAGGAGGAAGAAG/ AGGAATGGAGGGCCAAATC | | | | qRT-PCR |
| *OsIAA10* | TCTGATGTGCAGAAGGACAAG/ ATCCACCTTCCTTCCAATGAC | | | | qRT-PCR |
| *OsIAA11* | TACAAGAGCTACCCGGAGTT/ CCAGGTCATCTCTCAGCTTATTG | | | | qRT-PCR |
| *OsIAA12* | ACGCATCGTCTTCCTTCTTC/ TTCCATTTCTCGCTTCCTCTC | | | | qRT-PCR |
| *OsIAA13* | GGCTCCTCTCTCTTCTTCCA/ AATCAAGCTGCCTTCTCTTCTC | | | | qRT-PCR |
| *OsIAA14* | GGAGCACGTTCCAGTCTTC/ TACAGCCCTCCTCCCTTATT | | | | qRT-PCR |
| *OsIAA15* | GTGTCCTCTTGGTCTTGTATGT/ AGATCTTGGTGCTGCATGT | | | | qRT-PCR |
| *OsIAA16* | AGCCTTCTCCTTGCTTCAC / CCATCTCTCTCTCTCTGTCTCT | | | | qRT-PCR |
| *OsIAA17* | GGACCTCAAGACCTACAAGAAC/ CCTTGCGGCTCTCAGATAAG | | | | qRT-PCR |
| *OsIAA18* | CAGGTGAGAGGAAGAAAGGATG/ CAACCGACTACTGGAGGAATAG | | | | qRT-PCR |
| *OsIAA19* | GCCCTTAAGGACCAAGAGTATG/ CCTCTCATGATCCGCAGTTT | | | | qRT-PCR |
| *OsIAA20* | GCGGATATGTGAAGGTGAAGA/ GTGCCCATCCTCTTGGTTAG | | | | qRT-PCR |
| *OsIAA21* | GTGGACCTCAAGATGTACAAGAA/ CTCTCCCTGACTTCCCATTTG | | | | qRT-PCR |
| *OsIAA22* | CTCTGCAGAGCATGTTCCATG/ GCGGTCTCCCTCATTATCC | | | | qRT-PCR |
| *OsIAA23* | CCGAGGCCGTCAACTTATC/ GACAGACAGACAGACAGATCAAT | | | | qRT-PCR |
| *OsIAA24* | GTATGTGAAGGTGAGCATGGA/ CAGAGAAGCACTTGGTGAAGA | | | | qRT-PCR |
| *OsIAA25* | CAGTTTGGGAGAAGAGAATCCA/ AGGCTGTTCACATCACCTTC | | | | qRT-PCR |
| *OsIAA26* | CGTGTAAGAGGATGAGAGTGATG/ GATCGATGTCCAGGTTCAGTT | | | | qRT-PCR |
| *OsIAA27* | GACGACGTACATGCCAAGAG/ GTGTCGGGTTTCAGGACATTAT | | | | qRT-PCR |
| *OsIAA30* | ATTGGAAACTGTGGGTCTCAT/ CCTCGTAGGTTGGCACATATT | | | | qRT-PCR |
| *OsPIN1* | CTACCCATCGACCGATCCTA/ CTCACTCGGTGGCACTATTT | | | | qRT-PCR |
| *OsPIN1a* | GGTGAGGGTCGTCATGTTT/ CTTGGTTGCTTGGCTTCTTG | | | | qRT-PCR |
| *OsPIN1c* | CCTCTTGCCTCATCTGTGTC/ AGGAGGAGGAGAATAGGAGATG | | | | qRT-PCR |
| *OsPIN1d* | CGGTCGATTTCGATCCTTTCA/ GCATACGAAGCAAGGGAGTT | | | | qRT-PCR |
| *OsPIN2* | TTGGGTTGGCGGTACTAAAG/ TCCCAGTGTAGGATCGAAGT | | | | qRT-PCR |
| *OsPIN3a* | GCTCGCTGTGGAGAATAAGT/ GTATGGCCACTATGCACTAAGA | | | | qRT-PCR |
| *OsPIN5a* | AGCGATCAACCGCATTACA/ CCCACCCTATCATCTTCTTCTTC | | | | qRT-PCR |
| *OsPIN5b* | TGAGGTGGTGGAGGTTCT/ ACGAAGTCGAAGGTGAAGAAG | | | | qRT-PCR |
| *OsPIN5c* | CGAAACGCCAAGCATCATAG/ CCATGAACAAACCCATGCTAAA | | | | qRT-PCR |
| *OsPIN6* | GGTGAGTGTGAGATGGAAGATG/ CCTCCAAGAGAGTAGTGACAATG | | | | qRT-PCR |
| *OsPIN8* | TCAACACTTCCCAATACACTGA/ GGCTCTGGAGGACTACTATCT | | | | qRT-PCR |
| *OsPIN9* | TGGCCTCATCTGGTCTCTAA/ GCTATGAACGTCCCTGAAGAAA | | | | qRT-PCR |
| *OsPIN10b* | GCTCATCCTCACTACGGTTTG/ GCATCGGAGAGAATGGAGATTG | | | | qRT-PCR |
| *RGA1* | GCAAGACCCAGCCATTCAGAAAC/CTCTTGCATAAAGCACATCCTCCTTTG | | | | qRT-PCR |
| *RGB1* | GTGCTGCCGCACGCTTCA/CAACAGATTGGCCATTGGGTGC | | | | qRT-PCR |
| *RGG1* | GGTGTCAGCAGCATTGCAAGAG/GATCTTGCGGACCTTCAAACCATC | | | | qRT-PCR |
| *RGG2* | GGATGAACTGAACGAGCTTG/CTCTCCCAAGAAGCGTTCT | | | | qRT-PCR |
| *GS3* | CCTCAGCTACCTCTCC/GTTGCAGCAGCAGCTC | | | | qRT-PCR |
| *qPE9-1* | GTGCTGCGTGTGGAGATG/TGAGGAACGTGATCTCGCG | | | | qRT-PCR |
| OsPR1a | CCATACTAGCTAGTGATCTCTC/GATAACCTGCTCGACGAACT | | | | qRT-PCR |
| OsPR10a | GTGATCAGTTGCAACTAGCTAG/CATGAACGCCTTCCACAG | | | | qRT-PCR |
| OsActin | AGCAGCATGAAGATCAAGGTGGTC/CCTTGGCAATCCACATCTGCTG | | | | qRT-PCR |
| **Primers for generating DNA vectors and amplifying fragment including the target sequence (The red characters showed the restriction Joint sequence, the underline showed the homologous sequence with the vector sequences)** | | | | | |
| Primer name | | | Primer sequence | Experiment | |
| RGB1-g++ | | | TGATTGGTGTGCTGCCGCACGCTTCA | BGK01, CRISPR/Cas9 | |
| RGB1-g-- | | | AAACTGAAGCGTGCGGCAGCACACCA |
| RGB1_GUS_F | | | CCATGATTACGAATTCCTGCAGGCTATGGCTCATGTG | PCAMBIA1301, GUS | |
| RGB1_GUS_R | | | CTCAGATCTACCATGGGGTGTCGAGCAGCATCTGC |
| RGB1_GFP_F | | | TACAAATAAGAGCTCATGGCGTCCGTGGCGGA | pCambia1300-221, GFP | |
| RGB1_GFP_R | | | CATGATTACGAATTCTCAAACTATTTTCCGGTGTCCGCTG |
| RGB1_CAS9_check_F | | | TCCATGCCCGGATCCCAGT | For target sequence sequencing | |
| RGB1_CAS9_check_R | | | GGTACCAACCCAATCACCGAATC |
| PUV3_R_F | | | CTGGCGAAAGGGGGATGT | For Cas9 protein  separating | |
| gRNA_R5_R | | | ACGACCGGGTCACGCT |
| HYG_F_G | | | CGAGAGCCTGACCTATTGCAT |
| HYG_R_G | | | CTGCTCCATACAAGCCAACCA |
| **Probes for mRNA expression (The red characters showed the restriction Joint sequence, the underline showed the homologous sequence with the vector sequences)** | | | | | |
| Probe name | | Probe sequence | | Experiment | |
| In-RGB1-F | | GTGAGAACAGGGCACCAACT | | *In situ* hybridization | |
| In-RGB1-R | | CACCTCAGCGAGAAGTGTGT | |
