# Supplementary material for: The heterotrimeric G protein β subunit RGB1 is required for seedling formation in rice
Source: Rice (N Y). 2019 Jul 18;12:53. doi: 10.1186/s12284-019-0313-y (PMC6639528; doi:10.1186/s12284-019-0313-y)
Supplement: Supplementary file 8 — Table S3. Number of differentially expressed genes identified in the transcriptome analysis. (DOCX 13 kb) [file 12284_2019_313_MOESM8_ESM.docx]

**Table S5 The number of differentially expressed genes in transcriptome analysis**

| Comparison | p-value threshold | Number of differentially expression genes | Number of up-regulated genes | Number of  down-regulated genes |
| --- | --- | --- | --- | --- |
| CK-VS-*rgb1-2* | 0.05 | 9743 | 4624 | 5119 |
